# Signaling controversy and future therapeutical perspectives of targeting sphingolipid network in cancer immune editing and resistance to tumor necrosis factor-α immunotherapy

**DOI:** 10.1186/s12964-024-01626-6

**Published:** 2024-05-02

**Authors:** Olga A. Sukocheva, Margarita E. Neganova, Yulia Aleksandrova, Jack T. Burcher, Elena Chugunova, Ruitai Fan, Edmund Tse, Gautam Sethi, Anupam Bishayee, Junqi Liu

**Affiliations:** 1 Department of Hepatology, Royal Adelaide Hospital, Adelaide, SA 5000 Australia; 2 Institute of Physiologically Active Compounds at Federal Research Center of Problems of Chemical Physics and Medicinal Chemistry, Russian Academy of Sciences, Chernogolovka, 142432 Russian Federation; 3 Arbuzov Institute of Organic and Physical Chemistry, Federal Research Center, Kazan Scientific Center, Russian Academy of Sciences, Kazan, 420088 Russian Federation; 4 College of Osteopathic Medicine, Lake Erie College of Osteopathic Medicine, Bradenton, FL 34211 USA; 5 Department of Radiation Oncology, Cancer Center, The First Affiliated Hospital of Zhengzhou University, Zhengzhou, 450052 China; 6 Department of Pharmacology, Yong Loo Lin School of Medicine, National University of Singapore, Singapore, 117600 Singapore

**Keywords:** Tumor necrosis factor-α, Immunotherapy, Cancer drug resistance, Apoptosis, Sphingosine kinase, Sphingosine-1-phosphate, Sphingolipids

## Abstract

Anticancer immune surveillance and immunotherapies trigger activation of cytotoxic cytokine signaling, including tumor necrosis factor-α (TNF-α) and TNF-related apoptosis-inducing ligand (TRAIL) pathways. The pro-inflammatory cytokine TNF-α may be secreted by stromal cells, tumor-associated macrophages, and by cancer cells, indicating a prominent role in the tumor microenvironment (TME). However, tumors manage to adapt, escape immune surveillance, and ultimately develop resistance to the cytotoxic effects of TNF-α. The mechanisms by which cancer cells evade host immunity is a central topic of current cancer research. Resistance to TNF-α is mediated by diverse molecular mechanisms, such as mutation or downregulation of TNF/TRAIL receptors, as well as activation of anti-apoptotic enzymes and transcription factors. TNF-α signaling is also mediated by sphingosine kinases (SphK1 and SphK2), which are responsible for synthesis of the growth-stimulating phospholipid, sphingosine-1-phosphate (S1P). Multiple studies have demonstrated the crucial role of S1P and its transmembrane receptors (S1PR) in both the regulation of inflammatory responses and progression of cancer. Considering that the SphK/S1P/S1PR axis mediates cancer resistance, this sphingolipid signaling pathway is of mechanistic significance when considering immunotherapy-resistant malignancies. However, the exact mechanism by which sphingolipids contribute to the evasion of immune surveillance and abrogation of TNF-α-induced apoptosis remains largely unclear. This study reviews mechanisms of TNF-α-resistance in cancer cells, with emphasis on the pro-survival and immunomodulatory effects of sphingolipids. Inhibition of SphK/S1P-linked pro-survival branch may facilitate reactivation of the pro-apoptotic TNF superfamily effects, although the role of SphK/S1P inhibitors in the regulation of the TME and lymphocyte trafficking should be thoroughly assessed in future studies.

## Introduction

Remarkable innovations in cancer therapies have been achieved during the last few decades. Despite breakthroughs in treatment, cancer cells still manage to escape host immunity, survive, and progress towards treatment resistance in a subset of patients via multiple mechanisms, many of which remain unclear. One of the common reasons for inefficient cancer elimination is tumor immune evasion, the key mechanism that facilitates the failure of immune surveillance [1, 2]. During the efficient surveillance, cancer cells are designated for clearance if recognized as anomalous; and immune killing mechanisms are activated [1, 3]. The most successful endogenous death-initiating mechanisms rely on cytotoxic cytokines generated by natural killer (NK) T cells and/or phagocytes [1, 4, 5]. During acquisition of immune evasion strategies, the resistant cancer cell develops molecular tools which grant it immunity from NK-mediated cytotoxicity and cytokine attacks [6, 7], resulting in the activation of immunosenescence and promotion of an immunosuppressive tumor microenvironment (TME) [4].

A number of recently developed anticancer/immunotherapy pharmaceuticals aim to restore and strengthen internal surveillance capacity [1, 2]. The immune program relies on CD8+ and NK (CD3+ T lymphocytes) T cell subsets which can identify cancerous (as non-self) cells and delete them through complex clearance mechanisms, including release of inflammatory cytokines such as interferon-γ (IFN-γ) and tumor necrosis factors (TNF) [1, 8]. Initially defined as an endotoxin‐induced cytokine, TNF-α has demonstrated potent cancer-eradicating properties [9]. The ability to suppress cytotoxic cytokine signaling is a crucial survival adaptation for tumor cells. Notably, disruption of TNF-mediated cell death, normally initiated by CD8+ T cells, has been regarded as a major mechanism of immune evasion [1]. TNF-α is produced by the majority of immune cells, including macrophages, neutrophils, fibroblasts, keratinocytes, NK cells, T cells and B cells [5]. The cytokine activates apoptosis mainly through the death receptor (DR) pathway that is initiated by TNF-α receptor‐1 and -2 (TNFR1 and TNFR2) [10, 11]. TNF-α targets not only cancer cells, but also tumor-associated vasculature [6, 12, 13].

The internal tumor-related characteristics (cancer type and stage) and TME define the proapoptotic effects of TNF-α and its ability to inhibit tumor progression [6, 14]. For instance, human lymphoma is, generally, a TNF-sensitive type of cancer that demonstrates good immunotherapy response [15]. However, many solid tumors, including some breast malignancies, are intrinsically resistant to TNF-α effects. Cancer cell resistance to TNF-α cytotoxicity is a complex, multifactorial, and often unclear process. Several intrinsic factors and molecular mechanisms have been found responsible for the development of TNF-resistance, including mutation and downregulation of DR expression [10], activation of anti-apoptotic effectors (such as superoxide dismutase (MnSOD or SOD) [16, 17] and mitogen-activated protein kinase (MAPK)) [18]), diversion of nuclear transcription factor signaling (including nuclear factor kappa-light-chain-enhancer of activated B cells κB (NF-κB)) signaling [19]), and other pro-survival mechanisms. One of the survival pathways associated with anti-apoptotic and growth-promoting mechanisms is represented by the sphingolipid signaling axis [20, 21]. Sphingolipids are involved in the regulation of numerous intracellular mechanisms, both as mediators and effectors of signaling.

Besides regulation of cancer cell growth and metastasis, sphingolipids direct lymphocyte trafficking and cytokine responses, which are key factors in the resolution of inflammation [22–24]. The TNF-α/TNF receptors (TNFRs) network has been shown to trigger activation of sphingolipid signaling via sphingosine kinases 1 and 2 (SphK1 and SphK2). SphK1/2, “housekeeping” enzymes, are constitutively expressed and function to support the membrane metabolism in all cell types, including cancer and immune cells. These enzymes are responsible for the synthesis of sphingosine-1-phosphate (S1P), an established regulator of pro-survival machinery in multiple cancers. S1P and its transmembrane receptors (S1PRs) were found to be involved in the regulation of cytokine signaling and chronic inflammation [23–25]. Considering that sphingolipids, particularly those within the SphK/S1P/S1PR axis, are important effectors in the regulation of cancer cell survival and immune responses, these molecules may be considered as the key contributors to the development of immunotherapy resistance. However, the role of sphingolipids in the development of solid cancer resistance to immunotherapies and specifically to TNF-α-induced apoptosis remains to be clarified. Therefore, this review aims to discuss mechanisms of sphingolipid involvement in TNF-α-resistance in cancer cells and provide insights into the association of immune evasion with regards to SphK/S1P/S1PR axis.

## TNF superfamily signaling network: the cell death gatekeeping system

**Fig. 1:**
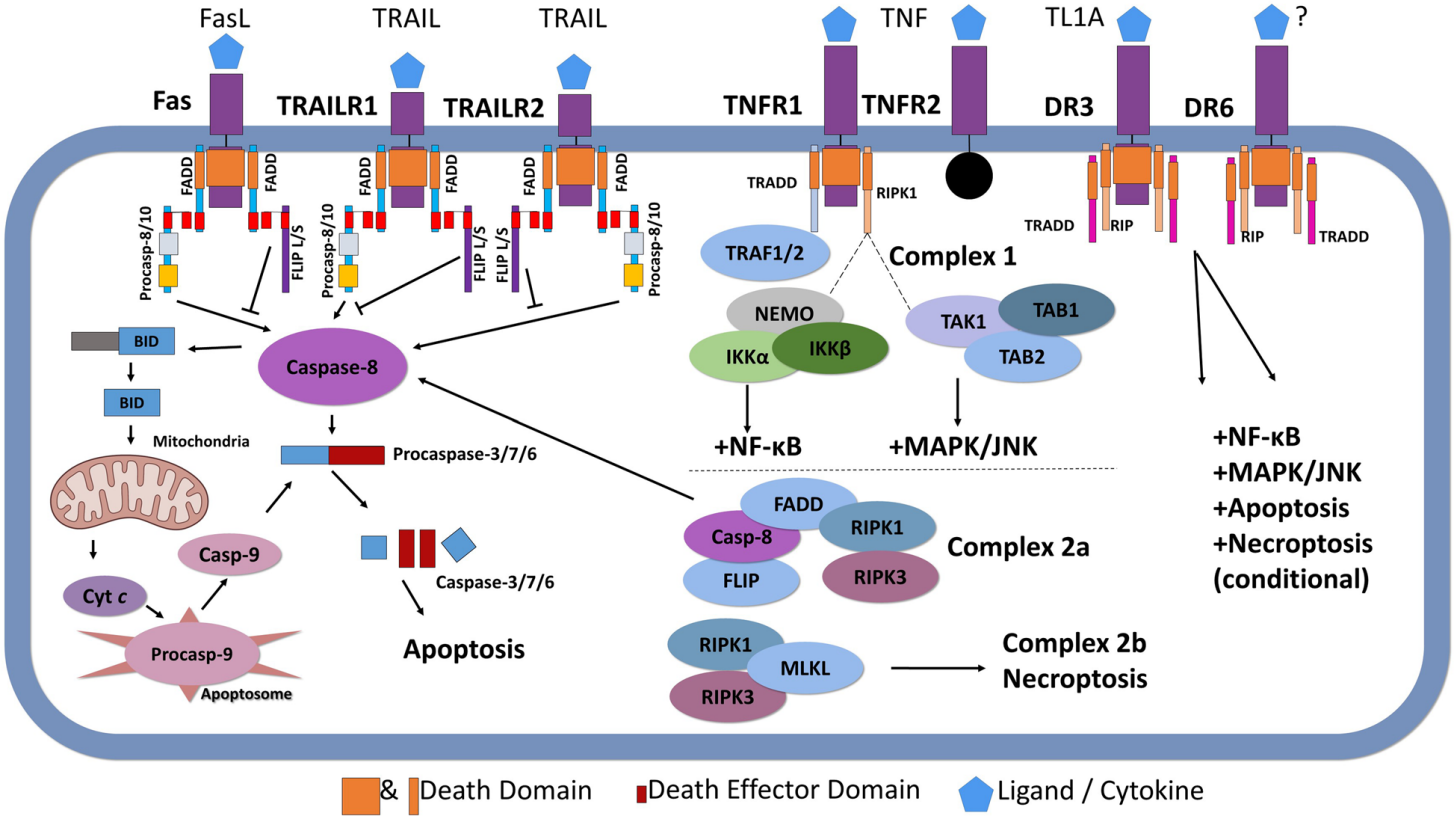
Death receptors (DR) and their ligands intracellular network. Ligands (FasL, TRAIL, TNF-α, TL1) can activate signaling cascades required for the activation of apoptosis and other complex cell responses. TNF-α/TRAIL/FasLs (and other ligands) bind the corresponding receptors (Fas, TRAIL-R1, and TNFR1) and activate apoptosis and necroptosis through interactions between death domains (FADD), TRADD adapter, and various caspases. Both TNFR1 and TNFR2 can trigger the classical NF-κB signaling. Binding of TNF to TNFR1 results in the formation of protein Complex I. Recruitment of IKKα/β through NEMO promotes activation of NF-κB and TAK1 induces MAPK signaling. Activation of the alternative NF-κB pathway is also possible via multiple mechanisms, leading to induction of survival effectors (MAPK and FLIP) which may counterbalance apoptosis (conditional). Complex I formation may also trigger pro-inflammatory and survival gene expression through these signaling pathways. Complex II formation results in the activation of caspase-8 and apoptosis. Should caspase-8 be inhibited, necroptotic cell death can occur instead. Abbreviations: FasL, Fas ligands; TRAIL, TNF-related apoptosis-inducing ligand; TNF-α, tumor necrosis factor-α; TL1, a novel TNF-like cytokine; TNFR1, TNF-α receptor 1; TNFR2, TNF-α receptor 2; FADD, FAS-associated death domain protein; TRADD, TNF receptor type 1-associated death domain protein; NF-κB, nuclear factor-κB; IKKα/β, IκB kinase α/β; NEMO, NF-κB essential modulator; TAK1, TGFβ-activated kinase 1; MAPK, mitogen activated protein kinase; FLIP, FLICE (FADD-like IL-1β-converting enzyme)-inhibitory protein

The TNF superfamily and TNFR network are crucial regulators of the extrinsic cell death (apoptosis) pathway and cancer cell surveillance [10, 26, 27]. The superfamily consists of signaling molecules (referred to as cytokines) that bind 29 corresponding receptors, including TNFRs [14, 27, 28]. Respective of what stimuli and/or receptors are involved, TNFRs can trigger not only several types of programmed cell death (apoptosis, necrosis, and anoikis), but also cell differentiation, migration, and proliferation [28–30]. The death-triggering mechanisms have been extensively reviewed [26, 28]. Several TNFR subtypes have been grouped according to the presence or absence of intracellular death domains (DDs, six α-helical fold fragment) [29]) (Fig. 1). The DD-containing subfamily includes TNFR1 (often named as p55, or DR1, or TNFRSF1A), Fas (CD95/TNFRSF6), DR3 (TNFRSF25), TNF-α-related apoptosis-inducing ligands (TRAIL) receptor 1 (TRAIL-R1, 1TNFRSF10A, DR4, CD261), TRAIL receptor 2 (TRAIL-R2, TNFRSF10B, DR5, CD262) [2, 4], DR6 (TNFRSF1), and EDAR [28, 31].

**Fig. 2:**
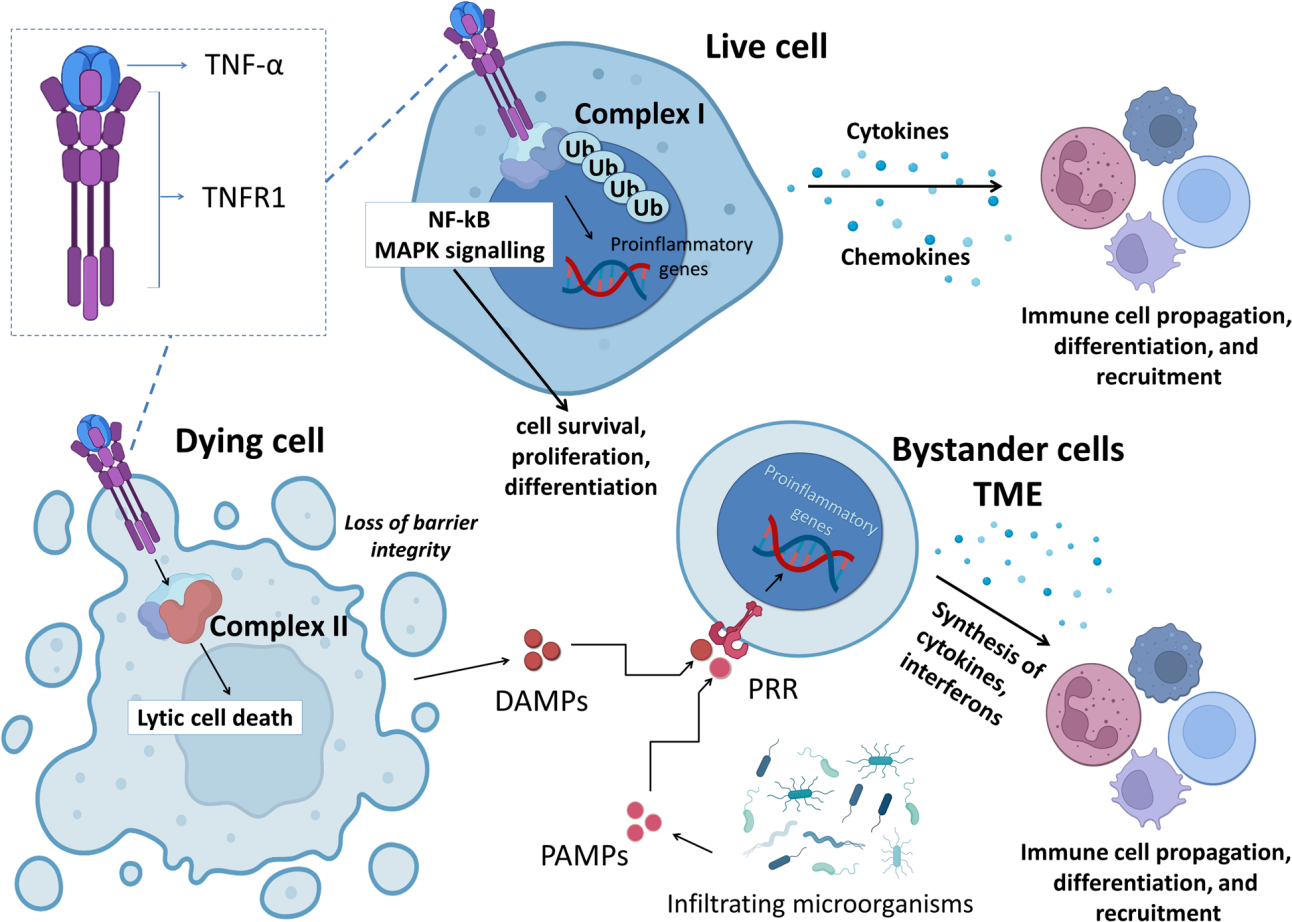
The dichotomy of TNF/TNFR effects is associated with activation of antagonizing effects, both promoting and counteracting cell death in immune cells. The resulting effect is defined by the active involvement of intracellular death machinery, which may be overruled by activation of pro-survival effectors. Both pathways lead to production of cytokines and propagation/differentiation of specific immune cells and their recruitment to the site of infection. Abbreviations: DAMPs, damage-associated molecular patterns; MAPK, mitogen-activated protein kinase; NF-κB, nuclear factor kappa B; PAMPs, pathogen-associated molecular pattern molecules; PRR, pattern recognition receptors; TNF-α, tumor necrosis factor-α; TNFR1, tumor necrosis factor-α (TNF-α) receptor-1; Ub, ubiquitin

The complexity of TNFR network is associated with the continuously expanding TNF superfamily of cytokines which presently includes 19 different ligands. TNF-α and TNF-β were identified several decades ago and have since been heavily studied. Other TNFR ligands, including homologous lymphotoxin (LT), ligands for Fas (or CD95), TRAIL (or APO2L), receptor activator of NF-κB ligand (RANKL), and osteoprotegerin ligand (OPGL) are relatively new members of this large family with poorly defined roles in cancer surveillance [14, 32, 33]. The full-length TNF-α is encoded by the TNF-α gene on human chromosome 6 [5]. TNF isoforms interact with TNFR1 and TNFR2 (defined as p75 or DR2) [11, 29, 32], leading to the formation of two signaling complexes I and II and cell death induction [3, 34] (Fig. 1). TRAIL can also induce apoptosis via binding to DR4 and DR5 in cancer cells [35, 36]. Fas and TRAIL receptors, the dual-signaling receptors, belong to the third DR subfamily with DD at their C-termini and TRAF recruitment domains at the opposing N-termini, providing them with the ability to activate NF-κB [34] (Fig. 2). Like the other members of the TNFR family, DR4/5 not only activate apoptosis, but can also regulate cell differentiation and proliferation [28, 30]. Functional TRAIL receptors (DR4/DR5) are widely expressed [37].

Activation of TNFR1 by its ligand is followed by receptor solubilization, membrane shedding, and binding of TNFR-associated death domain (TRADD), TNFR-associated factor 2 (TRAF2), receptor-interacting protein (RIP) kinase (RIPK) and transforming growth factor-β-activated kinase 1 (TAK1) proteins, leading to the activation of the classical NF-κB pathway [10, 32]. Recruited TRAF triggers ubiquitin ligase complexes (the upstream activator of NF-κB, activator protein 1 (AP-1)), p53 (tumor suppressor), and other transcription factors (Fig. 1) [10, 14, 32]. The internalized TNFR1 complex may also activate growth-regulating MAPK signaling effectors, including c-Jun N-terminal kinase (JNK) and p38 cascades [30, 33], ERK1/2 pathway, Fas-associated death domain (FADD)-like IL-1β-converting enzyme (FLICE) inhibitory protein (FLIP) [38], Bcl-2 (B-cell lymphoma 2) and Bcl-xL, and nitric oxide (NO) production [18, 38, 39]. Both TNFRs can also activate the phosphatidylinositol 3-kinase (PI3K)/Akt (protein kinase B) anti-apoptotic pathway in a TRAF2-dependent manner [30, 40, 41]. During DR-dependent activation of NF-κB and p53, TNF-α triggers extensive downregulation of XIAP, as well as cellular inhibitors of apoptosis protein-1 and -2 (cIAP1 and cIAP2), resulting in DNA fragmentation [42]. For instance, TNF-α was shown to induce apoptosis and DNA fragmentation within 24 h of treatment in MCF-7 mammary adenocarcinoma cells [42]. The quick (non-genomic) effects of TNF-α in MCF-7 cells start with the internalization of TNFR1. This results in the activation of caspase 8 and the cleavage of BID (a pro-death Bcl-2 family protein) [43]. Truncated tBID migrates to the mitochondria, causing activation of Bcl2 associated X protein (Bax)/Bcl-2 antagonist killer 1 (Bak), and release of cytochrome c (cyt *c*) [37, 43]. Following this, mitochondrial damage, and excessive production of reactive oxygen species (ROS) were observed [44]. Together with activated caspase 9, cyt *c* induces formation of the cytoplasmic apoptosome and irreversible propagation of apoptosis [37]. Death receptor (DR) activation can also inhibit expression of anti-apoptotic Bcl-2 and Bcl-xL [34, 39, 45].

**Fig. 3:**
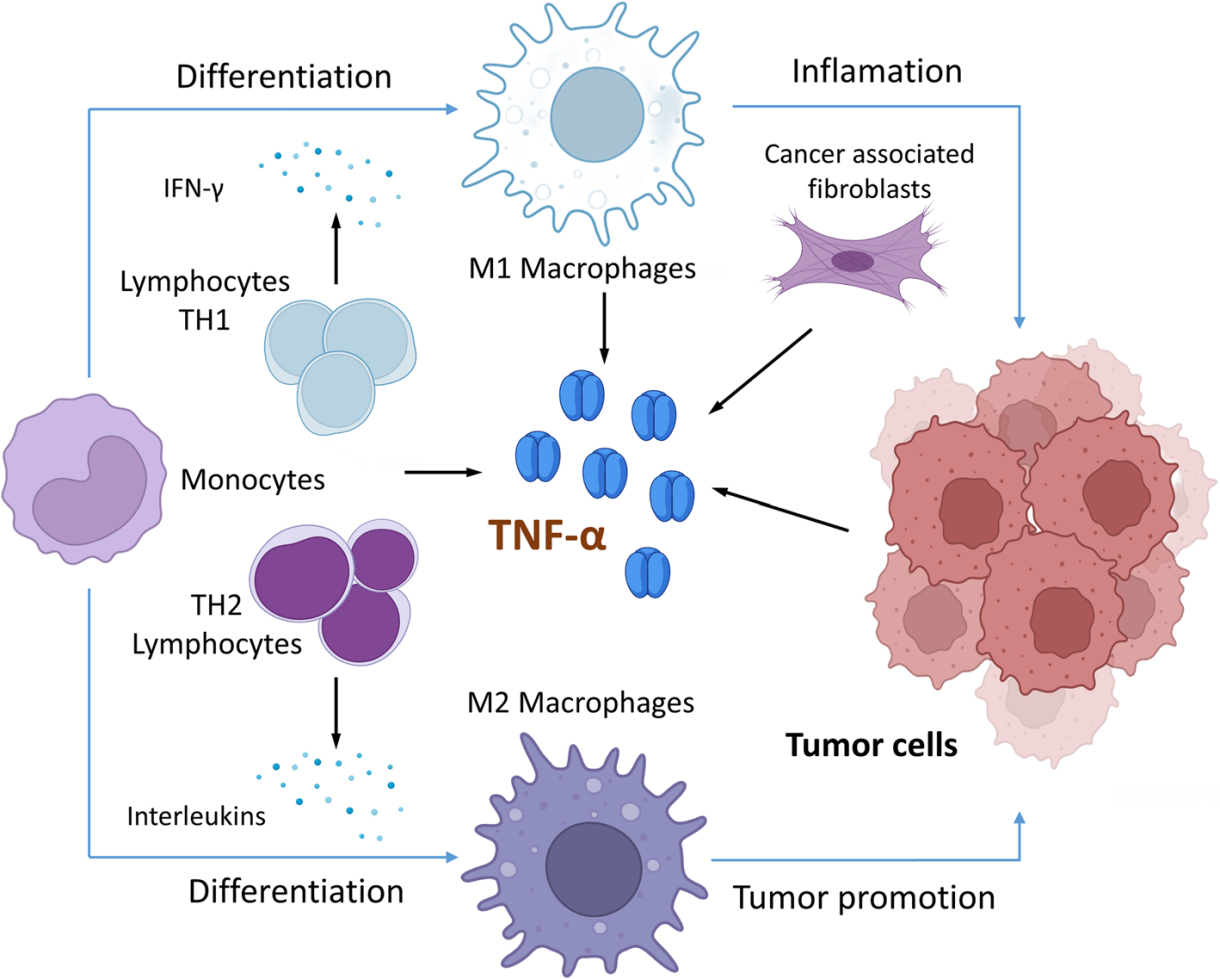
Involvement of TNF-α in the regulation of immune cell differentiation during inflammation and cancer progression. Promoting reprogramming of the TME, TNF-α was suggested to play central role as a connector of inflammation with cancer spreading. Abbreviations: INF-γ, Interferon‐γ; TH1, Type 1 T helper cell; TH2, Type 2 T helper cell; TNF-α, tumor necrosis factor-α

TNF expression is tightly regulated in normal cells and commonly induced during pro-inflammatory responses in various immune cells, fibroblasts, endothelial, and epithelial cells [46]. Macrophages and T cells are the major sources of secreted TNF-α, which targets all innate immunity cells responsible for pro-inflammatory effects in the TME, including differentiation of CD4+/CD8+ T cells [47, 48]. TNF-α may simultaneously activate both anti- and pro-apoptotic signals, that are required for the adaptation of immune system responses to dynamic intrinsic and/or extrinsic changes (Fig. 3). Using its non-apoptotic network, TNF-α induces differentiation of various immune cells, including monocytes/macrophages, microglia, Langerhans cells, and Kupffer cells [27]. Cancer spreading (metastasis) may also be triggered by TNF-α via the epithelial-to-mesenchymal transition (EMT) process [47, 49]. The activation of survival mechanisms was also noted during DR signaling in cancer cells and cells within the TME [5, 13, 40]. For instance, despite previously showing destruction of cancer-supporting blood vessels by TNF-α in cancer patients [12], intracellular TNFR1 signaling in endothelial cells activates two opposing pathways, one with pro-apoptotic effects [12], and another NF–κB-mediated pro-survival pathway [5, 13]. The multidirectional outcome of TNF-α signaling demonstrates the convoluted nature of this cytokine’s mechanistic actions, many of which remain largely unclear. The major anti-apoptotic effectors and pathways are discussed in this study, focusing on their connections to the sphingolipid network.

## Conventional mechanisms of TNF-α/TRAIL resistance in cancer cells

**Fig. 4:**
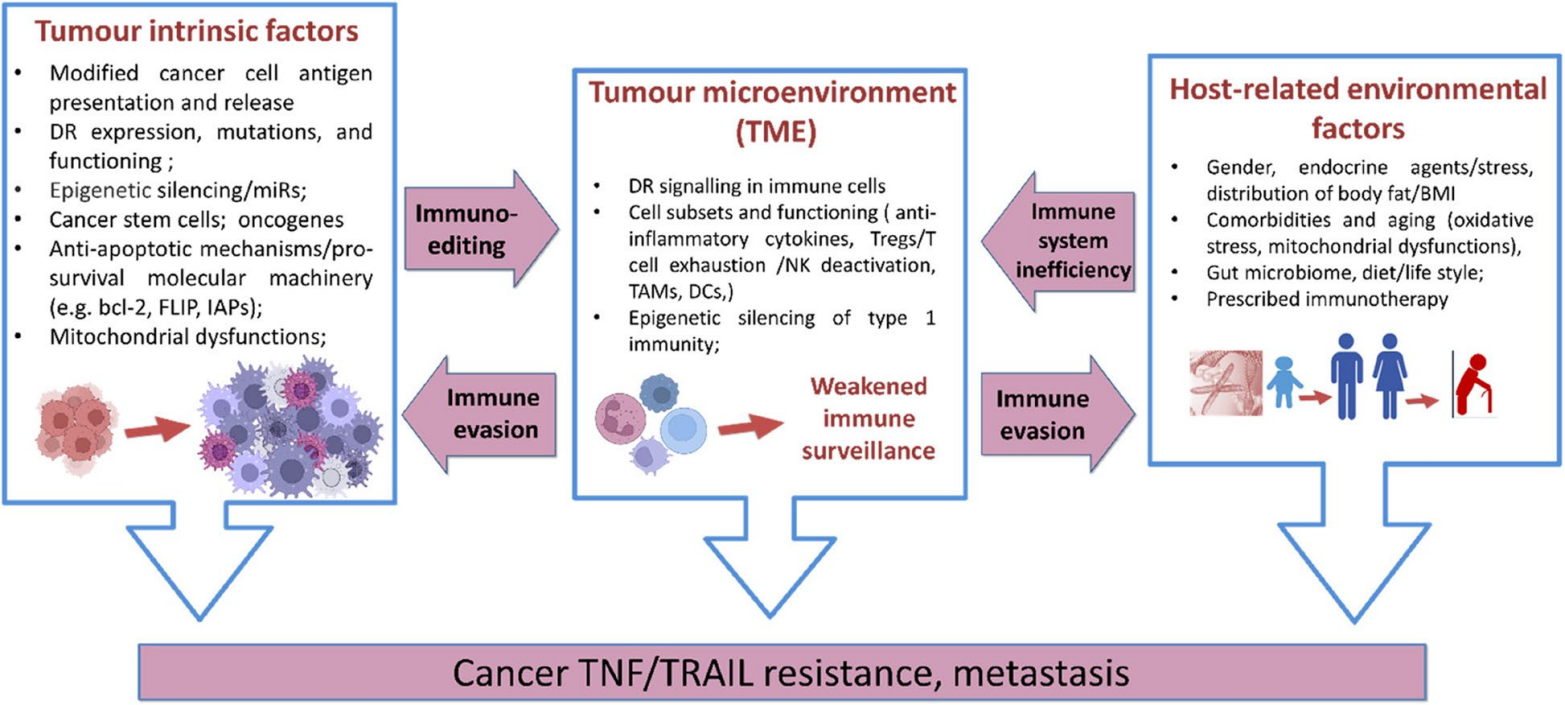
Interplay between cancer cell intrinsic factors, TME, and host-related factors that contribute towards the development of TNF/TRAIL resistance and metastasis. Abbreviations: Bcl-2, B-cell lymphoma 2; BMI, body mass index; DCs, dendritic cells; DR, death receptor; FLIP, FLICE (FADD-like IL-1β-converting enzyme)-inhibitory protein; IAPs, inhibitors of apoptosis; MiRs, micro ribonucleic acids; ROS, reactive oxygen species; TAMs, tumor-associated macrophages; TNF, tumor necrosis superfamily; TRAIL, TNF-related apoptosis-inducing ligand; Tregs, regulatory T cells

Cancer cells adapt to avoid recognition and elimination by the immune system (referred to as cancer immune evasion) [41]. Resistance to TNF-α/TRAIL is cancer-specific and can be mediated by several anti-apoptotic mechanisms. Among the most prominent TNF-α-resistance mechanisms are abnormal DR expression and functioning [50, 51], stoichiometry of the relevant ligand, heterozygous mutations and/or post-translational modifications of DRs and their ligands [50], mitochondrial dysfunctions, deficiency (lower expression or silencing) of key pro-apoptotic proteins/apoptosis pathway effectors (tumor intrinsic and host-related factors), low immunogenic capacity of immune effectors in the TME, and activation of complex pro-survival machinery [39, 51, 52]. Inhibition of TNF-α/TNFR-associated apoptosis was also detected in cells with dysregulated oxidative phosphorylation (OXPHOS) and/or abnormal expression/signaling of energy metabolism regulators, such as MnSOD [17, 53]. Considering the high level of cancer heterogeneity, complex resistance mechanisms may be present within one cancer tissue. Accordingly, the outcome of TNF/TRAIL-induced responses is determined by the relative contribution of the combined apoptotic signals, transmitted by DRs and their downstream targets, and pro-survival actions of cIAPs and other pro-survival effectors, including the growth-promoting and immunomodulatory components of the sphingolipid network. In TNF-α-resistant cancer cells, the combined pro-apoptotic signals are overwhelmed by pro-survival machinery, leading to cancer progression (Fig. 4). Notably, sphingolipid signaling contributes to many of the forementioned mechanisms. In this study, immune evasion-linked mechanisms of TNF/TNFR interactions within the sphingolipid network will be covered. The sphingolipid signaling axis is a recent addition to the list of cancer resistance-promoting modalities. Notably, sphingolipids were shown to be part of many different pro-survival and growth-stimulating networks, and thus may contribute to TNF-α resistance at multiple levels [21, 54].

## Sphingolipids as mediators, facilitators, and inhibitors of TNF-α-signaling

### SphK/S1P/S1PR axis: focus on growth-promoting and anti-apoptotic effects

A significant role in the regulation of sphingolipid signaling and metabolism by TNF-α has been demonstrated in multiple studies [13, 21, 55–57] (Table 1). In turn, both pro-survival and pro-apoptotic sphingolipids were implicated in the regulation of TNF-α/DR-induced effects.

**Table 1:** The SphK1/S1P/S1PR axis mediates and directs TNF-α signaling

**Disease or pathology; bioeffect**	**Main findings and biological mechanism involved**	**Cell type(s)/models**	**Position of SphK/S1P axis relative to TNF network**	**Reference**
Leukemia cell survival; apoptosis	SphK/S1P stimulates anti-apoptotic effectors, including ERK1/2. SphK1/S1P also blocks the apoptotic cascade upstream of the release of the mitochondrial apoptogenic factors, cyt *c*, and Smac/DIABLO.	Swiss 3T3 fibroblasts; human acute leukemia Jurkat, U937, and HL-60 cells	Downstream of TNF-α	[55, 56]
Pro-inflammatory effects of TNF-α in vasculature	S1P increases cerebral artery tone in rodent model of subarachnoid haemorrhage. Anti-apoptotic effects of SphK were demonstrated in endothelial cells.	Mouse olfactory cerebral resistance arteries; HUVECs	Downstream of TNF-α	[13, 58]
Bone growth	S1P/SphK1 stimulates proliferation and activation of osteoblasts. In osteoblasts S1P/SphK1 induces HSP27 and ERK1/2. TNF-α-induces expression of the c-fos and c-jun genes, which is also mediated by SphK1.	Osteoblast-like MC3T3-E1 cells	Downstream of TNF-α	[59, 60]
Allergic inflammation	S1P activates the MAPK pathway in BMMCs (mast cells) and promotes cytokine secretion. SphK1 inhibition results in reduction of mast cell-dependent airway hyperresponsiveness (lowered numbers of eosinophils and levels of the cytokines, including TNF-α).	BMMCs; murine model of allergic asthma	Upstream of TNF-α synthesis	[61, 62]
Neutrophil priming; inflammation	S1P enhances fMLP-stimulated superoxide production by neutrophils.	Human circulating blood neutrophils	Downstream of TNF-α	[63]
Prostate cancer; radiotherapy	Gamma-irradiation, together with TNF-α, induces apoptosis in prostate cancer cells via increased level of sphingosine (inhibition of SphK1).	LNCaP cells	Downstream of TNF-α	[64]
TNF-α/FasL-induced apoptosis; liver regeneration	TNF-α activates several anti-apoptotic factors, including SphK1, PI3K, and Akt. Protective anti-apoptotic effects of SphK1 were demonstrated.	Mouse or rat hepatocytes; bile duct ligation mouse model	Downstream of TNF-α	[65, 66]
Inflammation	SphK1 mediates TNF-α-induced stress fiber formation and activation of fibrosis.	Rat2 fibroblasts	Downstream of TNF-α	[67]
Anti-inflammatory effects of glucocorticoids and vitamin D	Glucocorticoid hormone and Vitamin D protects from TNF-α-induced apoptosis via activation of SphK1/S1P.	Human keratinocytes; fibroblasts	Dual: up- and downstream of TNF-α	[68, 69]
Brain cancer and inflammation	SphK1 overexpression potentiates the pro-inflammatory effect of TNF-α	C6 glioma cells	Downstream of TNF-α	[70]
Inflammation; apoptosis	TRAF2-binding motif of SphK1 was identified. The SphK-TRAF2 interaction results in the activation of the enzyme, which is required for TRAF2-mediated activation of NF-κB and apoptosis prevention. S1P was also defined as a co-factor for TRAF2 and associated NF-κB activation.	HEK 293 T cells; HUVEC; A7 cells	Downstream of TNF-α	[71, 72]
Early acute inflammatory response	SphK1 is involved in the regulation of neutrophil priming.	Blood neutrophils	Downstream of TNF-α	[73]
Cancer resistance to TNF-α; apoptosis	SPPase1 dephosphorylates S1P and increases the amount of sphingosine. SPPase1 mediates and increases TNF-α effects.	HEK293, MCF-7 cells	Upstream of TNF-α signaling	[74]
Activation of pro-inflammatory signaling	Sphk1/S1P mediates TNF-α signaling and induces both COX-2 activation and PGE2 secretion.	L929 fibroblast and A549 cancer cells	Downstream of TNF-α	[75]
Oxidative stress; glioma; apoptosis	TNF-α activates both neutral and acidic SMases which produce ceramide and induce apoptosis.	Human U-87 MG, U-373 MG, and U-251 MG glioblastoma cells	Downstream of TNF-α	[76]
Atherosclerosis	SphK1 mediates TNF-α-induced expression of inflammatory genes, such as MCP-1 and VCAM-1	Human aortic endothelial cells	Downstream of TNF-α	[77]
Anti-apoptosis; prosaposin	Anti-apoptotic (anti-TNF-α) effects of prosaposin are mediated by activation of SphK1 and ERK1/2.	U937 monocytic cells; PBMCs	Dual: up- and downstream of TNF-α	[78]
Neutropenia; peritonitis	SphK1 mediates the C5a-triggered inflammatory responses in vivo. Inhibition of SphK1 by DMS resulted in reduced neutropenia and improved peritonitis symptoms.	Male BALB/c mice (8–10 wk old); acute inflammation was induced by injection of human C5a	Dual: up- and downstream of TNF-α	[79]
Inflammation-related pancreatic cell death	TNF-α increases islet SphK activity and S1P biosynthesis, suggesting that S1P plays a role in the pathological response of pancreatic beta-cells to cytokines.	INS-1 insulinoma cells and isolated rat islets of Langerhans	Downstream of TNF-α	[80]
Lung cancer cell survival and inflammation	SphK1 mediates pro-survival TNF-α signaling in lung cancer cells.	A549 epithelial lung carcinoma cells	Downstream of TNF-α	[81]
Protective immunity and inflammatory responses	SphK1 negatively controls the inflammatory effects of Th1 cells by blocking the production of pro-inflammatory cytokines/chemokines.	DO11.10 CD4 + Th1 cells	Upstream of TNF-α synthesis	[82]
Apoptosis; breast cancer	Cathepsin B cleaves SphK1 in lysosomes. The decline in SphK1 occurs downstream of the initiator caspase but upstream of the effector caspase in TNF-α-treated cells.	MCF-7 breast cancer cells	Downstream of TNF-α	[83]
Macrophage-related inflammation; Mycobacterium tuberculosis	SphK1 is required for ERK1/2 activation in murine macrophages infected with mycobacterium. Overexpression of SphK1 confers resistance in macrophages to infection via enhanced generation of NO and expression of iNOS, pp38, and LAMP-2.	Murine BMMφ from 6–8 wk old BALB/c mice	Upstream of TNF-α synthesis	[84, 85]
Cardiovascular inflammation	TNF-α-induced ICAM-1 expression is reversed by addition of exogenous S1P.	HUVECs	Upstream (parallel) to TNF-α	[86]
Wound healing; extracellular matrix formation	SphK1 is required for TNF-α-mediated stimulation of MMP-1.	Human dermal fibroblasts	Downstream of TNF-α	[87]
Endovascular inflammation	SphK1/S1P/S1P1 and S1P3 receptor axis mediates TNF-α signaling to activation of Akt and eNOS.	HUVECs; HMVEC-C	Downstream of TNF-α	[88]
Diabetic retinopathy model	Inhibitors of SphK1 attenuate the effects of proliferative and inflammatory stimuli on retinal endothelial cells in vitro and in vivo (rats). In a mice model of DSS-induced ulcerative colitis, inhibition of SphK1 effectively diminished negative symptoms.	Human retinal endothelial cells; male Sprague–Dawley rats; male C57BL/6 mice; 293 T embryonic kidney cells. Diabetes was produced by intraperitoneal injection of streptozotocin.	Dual: up- and downstream of TNF-α	[89, 90]
Fumonisin B1 hepatotoxicity	Inhibition of iNOS expression diminishes generation of S1P and deprives liver cells from its protective effects.	Mice with targeted deletion of iNOS gene (Nos-KO)	Upstream of TNF-α synthesis	[91]
Anti-apoptosis	SphK1 mediates TNF-α-induced activation of Akt	1321N1 human astrocytoma cells	Downstream of TNF-α	[92]
Inflammatory responses; lipopolysaccharide (LPS) effects	LPS increases cellular levels of SphK1 mRNA and protein upstream of COX-2 and PGE2 synthesis and activation.	RAW macrophage	Dual: up- and downstream of TNF-α	[93]
Inflammation	SPP2 is highly upregulated by inflammatory stimuli in endothelial cells.	HUVECs	Downstream of TNF-α	[94]
Myogenesis	SphK1/S1P2 receptors mediate TNF-α-induced myogenesis (muscle regeneration).	C2C12 myoblasts	Downstream of TNF-α	[95]
Apoptosis	TNF-α stimulates the expression of adhesion proteins, including VCAM-1 and ICAM-1, via activation of neutral SMases and increases production of ceramide (which can be turned into S1P by ceramidase).	A549 epithelial lung carcinoma cells	Downstream of TNF-α	[96]
LPS-induced inflammation; astrogliosis	LPS induces the activation of retinal astrocytes (increases in GFAP expression) via JAK2/STAT3 and SphK/S1P axis. Aquaporin-4 was defined as an upstream regulator of SphK1.	Retinal astrocytes; primary astrocyte cultures isolated from aquaporin-4 (AQP4) + / + and AQP4-/- mouse embryos	Upstream of TNF-α synthesis	[97]
Inflammatory and allergic responses	S1P (SphK1 but not SphK2) induces degranulation of human mast cells.	LAD2 cells (closely related to CD34^+^-derived human mast cells; express FCRA receptors)	Upstream of TNF-α synthesis	[98]
Collagen-induced arthritis (model)	Inhibition of SphK1 significantly reduces articular inflammation. SphK1 siRNA downregulates serum levels of TNF-α. Ex vivo analysis demonstrated suppression of collagen-specific pro-inflammatory/Th1 cytokine release in SphK1 siRNA-treated mice. Mice with SphK2 siRNA develop more aggressive disease with higher serum levels of TNF-α and other pro-inflammatory cytokines.	Male DBA/1 mice at 8–10 wk old; murine lymph node cell cultures	Upstream of TNF-α synthesis	[99]
LPS-induced lung injury	Overexpression of SphK1 (delivered by adenoviral vector) protected SphK1(-/-) mice from lung injury (reduced TNF-α release), although SphK2 aggravated it.	SphK1 knockout (SphK1(-/-)) and wild-type (WT) mice	Upstream of TNF-α synthesis	[100]
Acute peritonitis	The anti-inflammatory activity of resveratrol is mediated via inhibition of SphK1.	C5 anaphylatoxin (C5a)-stimulated peritonitis in mice	Upstream of TNF-α synthesis	[101]
DSS-induced colitis	SphK1 mediates induction of COX-2 by TNF-α in vivo.	SphK1(-/-) mice	Downstream of TNF-α	[102]
Rheumatoid arthritis, endothelial inflammation	S1P synovial fluid levels were significantly higher in patients with rheumatoid arthritis. SphK1 mediates TNF-α signaling towards pro-inflammatory responses in vasculature.	HUVECs; human synovial fluids from patients with rheumatoid arthritis	Dual: up- and downstream of TNF-α	[103]
Synovial inflammation and joint erosion (TNF-α-induced arthritis)	Mice lacking SphK1 possess less articular COX-2 protein and fewer synovial Th17 cells. SphK1 mediates and promotes TNF-α-induced inflammatory arthritis via impacting synovial inflammation. Genetic inhibition of SphK2 did not impact the severity of arthritis, while pharmacologic inhibition of SphK2 by ABC294640 led to more severe arthritis.	Transgenic TNF-α mice with spontaneous inflammatory arthritis, crossed with SphK1 null mice (SphK1(-/-)), on the C57BL6 genetic background	Dual: up- and downstream of TNF-α	[104, 105]
Neuroinflammation; neural tissue degeneration	Inhibition of SphK1 signaling results in decreased TNF-α expression in LPS-activated microglia. Exogenous S1P recovers TNF-α level in microglia.	BV2, a murine microglial cell	Upstream of TNF-α synthesis	[106]
Pro-inflammatory effects of TNF-α	TNF-α stimulated SphK1 activity and expression.	HEK293	Downstream of TNF-α	[107]
Inflammatory and autoimmune disorders	SphK1 mediates TNF-α-induced activation of the integrin α5-β1.	HUVECs	Downstream of TNF-α	[108]
Pathogenesis of postoperative ileus	Pro-inflammatory effects of S1P were demonstrated in intestinal muscles. SphK1 mediates TNF-α and the LPS-induced activation of NF-κB in RISM cells.	Primary cultured rat intestinal smooth muscle (RISM) cells	Downstream of TNF-α	[109]
Cochlear blood flow; ischemic hearing loss	TNF-α reduces cochlear blood flow via activation of vascular SphK1 signaling.	Patients with hearing loss	Downstream of TNF-α	[110]
Inflammation; airway epithelial barrier function	SphK1 stimulates the expression of mucin MUC5AC in cells stimulated with TNF-α.	HBE16 airway epithelial cells	Downstream of TNF-α	[111]
Hyperalgesia; pain management	S1P contributes to the development of hyperalgesia via the S1P1.	Male Sprague Dawley rat model with intraplantar injection of C_2_-ceramide	Downstream of TNF-α	[112]
DENV infection	DENV reduced level SphK activity leading to reduced TNF-α pro-apoptotic signaling. SphK/S1P also regulates IL-6 synthesis.	DENV-2-infected monocyte-derived macrophages or HEK-293 cells	Downstream of TNF-α	[113]
Atherosclerosis; vascular inflammation	The S1P3 receptor promotes the chemotactic effect of S1P in macrophages, inflammatory monocyte and macrophage recruitment, and alters smooth muscle cell behaviour in vitro and in vivo.	S1P3(-/-)/ApoE(-/-) double knockout mice; bone marrow-derived S1P3-deficient macrophages	Dual: up- and downstream of TNF-α	[114]
Diabetes	TNF-α enhances myogenic tone (vasoconstriction) by enhancing S1P levels. S1P1 receptors provide podocyte-specific protection against kidney inflammation and injury.	Human skeletal muscle resistance arteries; C57BL/6N diabetes mouse model (high-fat diet plus streptozotocin); immortalized podocytes	Dual: up- and downstream of TNF-α	[115, 116]
Inflammation during liver transplantation	S1P hepatic concentration grew after liver transplantation along with increases in levels of pro-inflammatory cytokines. SphK2 inhibitor blocked the observed effect.	Rat model of liver transplantation; Inbred male Lewis rats	Dual: up- and downstream of TNF-α	[117]
Hepatotoxicity; liver regeneration	S1P downregulates the ATPase by inhibiting both JNK and NF-κB.	HepG2 cells	Downstream of TNF-α	[118]
Cancer chemoresistance	Pharmacological inhibition of SphK1/2 by SKI-II induces apoptosis in TNF-α-resistant lung cancer cells through modulation of the NF-κB pathway.	MCF-7TN-R and MDA-MB-231 breast cancer cells	Dual: up- and downstream of TNF-α	[119]
Heart failure; myocardial infarction; SphK1 inhibitor PF543	TNF-α downregulates cystic fibrosis transmembrane conductance regulator which is a critical regulatory site for S1P signaling in the mouse model of heart failure. TNF-α induces cerebral artery vasoconstriction and decreases cerebral blood flow under the control of SphK1/S1P. Treatment with SphK1 inhibitor PF543 improved the myocardial structure and function.	C57BL6 mice myocardial infarction model; CFTR knockout mice (*CFTR* − / −); *Sphk1*^−/−^ and *Sphk2*^−/^ KO mice; murine vascular smooth muscle cells; mouse cerebral arteries; Sprague Dawley rats; H9c2 cells	Downstream of TNF-α	[120–122]
Acute pancreatitis	The expression of SphK1/S1P3 and SphK1 activity are increased in peripheral immune cells in the early stage of pancreatitis.	Peripheral neutrophils, monocytes/lymphocytes; acute pancreatitis (humans)	Upstream (parallel) of TNF-α synthesis	[123]
Oxidative stress; neurodegeneration; neuronal survival	TNF-α-mediated activation of Mg(2+)-nSMase and NOX in neuronal cells results in the production of the neurotoxic intermediates ceramide and ROS, damages SphK1 signaling, and accelerates neurodegeneration.	SH-SY5Y human neuroblastoma	Downstream of TNF-α	[124]
Apoptosis; inflammation; isoflurane anti-inflammatory effects	SphK1 demonstrates anti-apoptotic properties and modulates isoflurane’s beneficial effects in endothelial cells and brain injury model in vivo.	EA.hy926 umbilical vein endothelial cells; male CD-1 mice with subarachnoid hemorrhage/brain injury	Upstream of TNF-α	[125, 126]
Sepsis; hyper-inflammation	Increased SphK1 mRNA is observed in endotoxemic aged rats (LPS-treated Kupffer cells). The effect correlated with a significant increase in TNF-α mRNA levels in the liver.	Endotoxemia model of sepsis in aged rats; hepatic tissues	Upstream (parallel) of TNF-α synthesis	[127]
Anti-inflammatory mechanisms	SphK1 inhibits production of RANTES through activation of p38 MAPK.	HeLa and A549 cells; mouse embryonic fibroblasts	Downstream of TNF-α	[128]
Breast cancer; fibroadenomas	SphK1 is positively expressed in breast tumors but absent in fibroadenomas. TNF-α stimulates expression of SphK1, which is linked to decreased expression of E-cadherin (promoted metastasis).	MCF-10A, MCF-7 cells	Downstream of TNF-α	[129]
Obesity; fat cell metabolism; inflammation	SphK1 is involved into adipocyte-related inflammation and cytokine secretion.	3T3-L1 adipocytes; RAW264.7 macrophages	Upstream of TNF-α synthesis	[130]
Atopic dermatitis; Pseudomonas aeruginosa	A *Pseudomonas aeruginosa*-derived neutral ceramidase activates S1P/S1P receptors which stimulate secretion of TNF-α in keratinocytes.	Normal human epidermal keratinocytes	Upstream of TNF-α synthesis	[131]
Stroke; cerebral ischemia/reperfusion (I/R)	Stroke model in mice results in upregulation of TLR2 and Sphk1 expression in microglial cells. TLR2 or SphK1 blockade also inhibits synthesis of TNF-α. Inhibition of S1P receptors by FTY720 reduces stroke-related damage.	C57BL/6 mice; male ICR mice; cerebral artery occlusion model	Upstream of TNF-α synthesis	[132, 133]
Acute LPS-induced liver failure	Inhibition of SphK1 ameliorates liver failure and reduces inflammation.	BALB/c mice model of liver failure; PBMCs	Upstream of TNF-α synthesis	[134]
Pulmonary infection with Cryptococcus neoformans	Primary neutrophils from SphK1(-/-) mice showed impaired antifungal activity. High TNF-α was reported (in the mice infected with C. neoformans) and was dependent on the SphK1/S1P pathway. SphK1/S1P pathway promotes host defence against *C.* neoformans infections by regulating TNF-α levels.	Immunocompetent mice (CBA/J and C57BL6/J); Tgε26 (an isogenic strain of strain CBA/J lacking NK cells), and SphK(-/-) (an isogenic strain of C57BL6/J, lacking SphK1)	Upstream of TNF-α synthesis	[135]
Sepsis; apigenin	Apigenin induces activation of SphK1 and protected cardiomyocytes from inflammation-related damage and apoptosis.	LPS-induced sepsis in Wistar rats; rat embryonic heart-derived myogenic cell line H9c2	Upstream of TNF-α	[136]
Atherosclerosis	Prolonged lowering of plasma S1P (inhibition of SphK1) results in pro-atherogenic effects in rodents.	low-density lipoprotein receptor deficient (LDL-R-/-) mice	Upstream of TNF-α synthesis	[137]
Macrophage chemotaxis; periodontitis linked to Aggregatibacter	Bacterial infection increases SphK1 expression. Low levels of S1P promote BMM chemotaxis. SphK inhibition decreases infiltration of periodontal tissues with leukocytes (lowered inflammation).	Murine BMMs; SphK1 KO mice	Upstream of TNF-α effects	[138]
Acute liver failure	The C5a/C5aR axis upregulates SphK1 expression via p38 MAPK.	BALB/c mice; LPS injection	Upstream of TNF-α synthesis	[139]
Animal model of acute liver failure (ALF) induced by RHDV	S1P/S1P1 levels are significantly elevated following RHDV infection. Melatonin administration inhibits the effect and suppresses immunoreactivity against RHDV viral VP60 antigen in the liver. SphK1/S1P system is activated in parallel to viral replication.	Rabbits; haemorrhagic disease virus (RHDV)	Downstream (and/or parallel) to TNF-α signaling	[140]
Experimental Chagas disease cardiomyopathy	SphK1 mediates TNF-α-induced activation of lymphocytes in cardiac inflammation model in rodents.	Male C57BL/6 mice infected with myotropic Colombian *T. cruzi*	Downstream of TNF-α	[141]
Breast cancer	High *SPHK1* expression and increased production of S1P in the blood during BC development was observed. Non-classical monocytes in BC had increased levels of S1PR2 and S1PR3, a profile that is abrogated under chemotherapy.	PBMCs from breast cancer patients with/ without chemotherapy; granulocytes, and monocytes	Dual: up- and downstream of TNF-α	[142]
IBD	Inhibition of SphK1 reduced the expression of pro-inflammatory markers and reduced neutrophil infiltration in colon tissue.	DSS murine model for IBD	Upstream of TNF-α synthesis	[143]
Ulcerative colitis; cycloastragenol; protocatechuic acid	Cycloastragenol reduces expression of SphK1, TNF-α secretion, and improves colitis. Protective effects of protocatechuic acid in mouse colitis model are also mediated by SphK1/S1P.	Acetic acid (intracolonic)-induced colitis in Sprague Dawley rats; TNBS-induced colitis in BALB/c mice	Upstream of TNF-α synthesis	[144, 145]
Joint arthroplasty	SphK2 is involved in macrophage activation and TNF-α release.	RAW264.7 macrophages	Upstream of TNF-α synthesis	[146]
Erythropoiesis; myelopoiesis anaemia; hCD34^+^ hematopoietic cells	TNF-α/neutral SMase/ceramide pathway inhibits erythropoiesis to induce myelopoiesis. The process requires inhibition of SphK1/S1P production. S1P restores erythroid differentiation.	Human CD34^+^ hematopoietic stem/progenitor cells	Dual: up- and downstream of TNF-α	[147]
Pathogenesis of fructose-induced NAFLD; effects of CGA (and/or Telmisartan)	Increased level/activation of SphK1/S1P/S1P1 and S1P3 (upstream of NF-κB activation) in NAFLD rats was observed. Telmisartan/CGA decreases these effects, indicating that inhibition of angiotensin II and the SphK1/S1P axis is an effective anti-inflammatory tool. Telmisartan is the angiotensin II receptor and ACE blocker, and a strong antioxidant.	Male Wistar rats; NAFLD rat model	Upstream of TNF-α synthesis	[148]
Hepatic I/R injury; apoptosis; necrosis; oxidative stress	I/R-associated inflammation is alleviated in SphK1 KO mice. Lowered expression of S1P1, reduced phosphorylation of NF-κB p65 and STAT3, inflammation (IL-1β, IL-6, TNF-α), and oxidative stress were detected.	SphK1 KO wild type mice	Upstream of TNF-α synthesis	[149]
ALD mice models; liver organoids; cirrhosis; HCC	SphK2 deficient (SphK2^−/−^) mice on alcohol diet exhibit a greater degree of liver injury and hepatic lipid accumulation. SphK2 expression levels are downregulated in the livers of human patients with alcoholic cirrhosis and HCC.	SphK2^−/−^ mice; intestinal organoids; human patients with alcoholic cirrhosis	Upstream of TNF-α synthesis	[150]
Saturated fatty acids (SFA); inflammation	SFA (myristate) activates SphK1 and triggers expression of TNF-α in colon cells.	Intestinal epithelium (IEC6) cells, C57BL/6 male and female mice; HFD study	Upstream of TNF-α synthesis	[151]
Lung cancer	S1P mediates Toll-like receptor 9 (TLR9)-induced release of the pro-inflammatory cytokines, including TNF-α.	Lung adenocarcinoma A549 cells	Upstream of TNF-α synthesis	[152]
Apoptosis; cancer progenitor cell growth and division	TNF-α inhibits mammosphere formation and induces S1P3 internalization and degradation. TNF-α-treated MCF-7 cells demonstrated increased apoptosis and no nuclear localization of SphK1/S1P3, suggesting that TNF-α can inhibit nuclear translocation of SphK1/S1P3.	MCF-7 breast cancer cells; mammospheres (enriched with BC progenitor cells)	Dual: up- and downstream of TNF-α	[153]
Muscle dysfunction	SphK1 mediates TNF-α-induced myotube atrophy and autophagy.	Skeletal muscle C2C12 myotubes	Downstream of TNF-α	[154]
Acute liver failure	Deletion of SphK1 (not SphK2) decreases liver damage, hepatic apoptosis, serum alanine aminotransferase levels, and mortality rate in mice. LPS-induced TNF-α level is suppressed in SphK1-deleted macrophages, whereas IL-10 expression is enhanced (anti-inflammatory phenotype).	SphK1^−/−^ mice model with D-galactosamine GalN/LPS-induced liver damage	Upstream of TNF-α synthesis	[155]
Preeclampsia (PE)/inadequate placental function	Placental SphK1 is increased in preeclampsia. Inhibiting SphK1 alone decreases TNF-α release and reverses TNF-α-dependent decreases in IL-10 release.	Human placenta samples; Placental chorionic villi (explant culture)	Dual: up- and downstream of TNF-α	[156]
Hypoxia; NK cytotoxicity; NK resistance	SphK1 knockdown reverses hypoxia-induced cell resistance to NK cell killing.	Bladder cancer cells were co-cultured with NK cells	Dual: up- and downstream of TNF-α	[157]

*Abbreviations*: *ACE* Angiotensin converting enzyme, *ALD* Alcoholic liver disease, *BC* Breast cancer, *BMM* Bone marrow-derived monocyte/macrophage, *BMMCs* Bone marrow-derived mouse mast cells, *BMMφ* Bone marrow-derived macrophages, *CFTR* Cystic fibrosis transmembrane conductance regulator, *CGA* Chlorogenic acid, *COX-2* Cyclooxygenase-2, *DENV* Dengue virus, *DIABLO* Direct Inhibitor of Apoptosis-Binding protein with LOw pI, *DSS* Dextran sodium sulfate, *eNOS* Endothelial Nitric Oxide Synthase, *ERK* Extracellular-signal-regulated kinase, *fMLP* N-Formylmethionyl-leucyl-phenylalanine, *GFAP* Glial fibrillary acidic protein, *HCC* Hepatocellular carcinoma, *HMVEC-C* Human cardiac microvascular endothelial cells, *HSP27* Heat shock protein 27, *HUVECs* Human umbilical vein cells, *IBD* Inflammatory bowel disease, *ICAM-1* Intercellular adhesion molecule-, *ICR* Institute of Cancer Research, *IL* Interleukin, *iNOS* Inducible nitric oxide synthase, *JAK2* Janus kinase 2, *JNK* c-Jun N-terminal kinase, *KO* Knockout, *LAMP-2* Lysosomal associated membrane protein-2, *LPS* Lipopolysaccharides, *MCP-1* Monocyte chemoattractant protein-1, *MMP-1* Matrix metalloproteinase 1, *mRNA* Messenger ribonucleic acid, *NAFLD* Non-alcoholic fatty liver disease, *NF-κB* Nuclear factor kappa B, *NK* Natural killer, *PBMCs* Peripheral blood mononuclear cells, *PI3K* Phosphatidylinositol 3-kinase, *RANTES* Regulated upon activation, normal T cell expressed and secreted (also known as CCL5), *RHDV* Rabbit haemorrhagic disease virus, *S1P1* Sphingosine 1-phosphate receptor 1, *S1P3* Sphingosine-1-phosphate receptor 3, *siRNA* Small interfering ribonucleic acid, *Smac* Second mitochondria-derived activator of caspase, *SMases* Sphingomyelinases, *SphK* Sphingosine kinase, *SPPase1* Sphingosine-1-phosphate (S1P) phosphatase 1, *STAT3* Signal transducer and activator of transcription 3, *TNBS* 2,4,6-trinitrobenzene-sulfonic acid, *TRAF2* Tumor necrosis factor (TNF) receptor associated factor-2, *VCAM-1* Vascular cell adhesion molecule-1

**Fig. 5:**
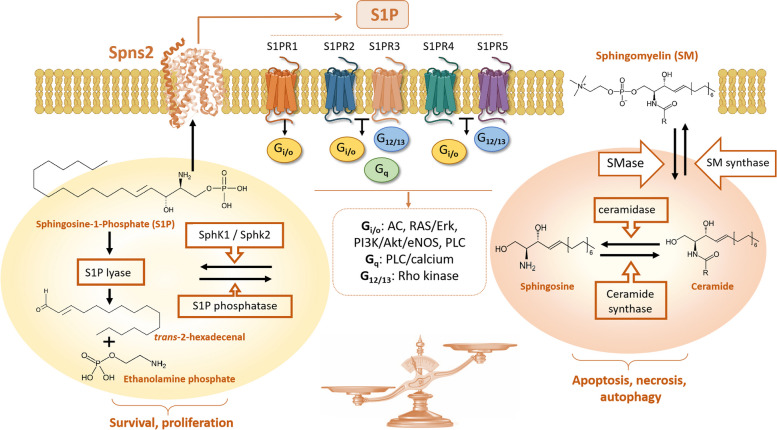
The sphingolipid signaling pathway. Various sphingolipids molecules (second messengers) can be derived from the membrane lipid sphingomyelin by sphingomyelinase (SMase) and metabolised by a “rheostat”-forming network which regulate homeostasis. Accumulation of ceramide and sphingosine can tip the balance towards apoptosis and other types of cell death. Activation of SphK1/2, production of S1P (and activation of S1P receptors), and/or S1P degradation by S1P lyase to hexadecenal and ethanolamine phosphate result in pro-survival and growth-promoting effects. Sphingomyelin membrane content can be restored through activation of sphingomyelin synthase (SM Synthase), which can also help to minimise the content of ceramide. The amount of sphingosine can be increased via inhibition of SphK1/2 and or through activation of S1P phosphatase

For instance, activation of apoptosis by DRs has previously been shown to downregulate SphK1 protein expression and activity via proteasomal degradation [158, 159]. Sphingolipids are not only structural components of all biological membranes, but also signaling and regulatory molecules. The variety of sphingolipid family members and their functions have been reviewed previously [160, 161]. The accumulation of apoptosis-inducing members, ceramide and sphingosine, was noted during DR-signaling [54, 160, 161] (Fig. 5) (Table 1). Key enzymes responsible for ATP-dependent metabolism of sphingosine and generation of S1P include SphK isoforms (SphK1 and SphK2), which are found in cytoplasmic, mitochondrial, and nuclear compartments [57, 153, 161, 162]. Activation of SphK1 and S1P synthesis are responsible for growth-stimulating and pro-survival effects in normal and cancer cells [21, 54, 57, 163]. The role of SphK1 will be considered as a counterbalancing anti-apoptotic force for DRs in this review [61, 164]. Both SphK1 and SphK2 were suggested to mediate numerous cellular responses to external stimuli and stress [21, 54, 151, 153]. Notably, SphK2 was reported to suppress proliferation and facilitate propagation of apoptosis, thus playing an opposite role to SphK1, although this hypothesis remains to be confirmed [25, 165]. The roles of SphK2 in DR signaling and propagation of apoptosis have been discussed previously [166].

S1P is a multifunctional messenger which can bind both intracellular targets and membrane-located (extracellular) receptors. Paracrine-, blood-, or lymph-released S1P binds transmembrane S1P receptors (G-protein coupled S1PRn (*n* = 1–5)), which are the established effectors of growth and survival [21, 167] (Fig. 5). S1PR1 is abundantly expressed in all cell types, including large variety of immune cells [168, 169], indicating the high importance of this receptor for the regulation of vital cell functions. The receptor signals via G_i/o_ heterotrimeric proteins which may inhibit adenylyl cyclase and activate potassium channels [170].

S1PR2 is also ubiquitously expressed [168, 171], although the receptor signaling remains less investigated. Notably, S1PR2 was shown to inhibit colorectal cancer tumorigenesis [172]. The activation of S1PR2 or S1PR3 was linked to the activation of G_i/o_, G_q_, and G_12/13_, suggesting the potential activation of large variety of downstream effectors [173]. Aside from normal cell types, S1PR3 is highly expressed in various cancer cells and was shown to stimulate cancer progression [21, 153, 168]. It is common to observe the co-expression of different S1P receptors, especially presence of S1PR1 and S1PR3 within one cell type which may indicate cooperation of signaling among the receptors [174]. In comparison to S1PR1 and S1PR3 effects, S1PR4 was found to be growth-inhibitory in some immune cells [175], while its role in the lymphocyte trafficking and expansion was extensively discussed [169]. The receptor may regulate the cytotoxicity of T cells towards cancerous tissues [176], although downstream signaling pathways of S1PR4 remain largely unclear. S1PR5 was also shown to regulate T cell subtype maturation and functions [177]. G_i/o_ and G_12/13_ were shown to transmit S1PR4 and S1PR5 signals in normal and malignant cells [178]. The expression of S1PRs in both cancer and immune cells represents a debatable phenomenon which was recently reviewed [169]. To complicate the problem, the level of S1PR expression may vary during morphogenesis, cell growth and differentiation [153]. The growth-promoting and/or pro-carcinogenic role of S1PR1 and S1PR3 seems confirmed. However, current knowledge does not provide unequivocal answer about the role of different S1PRs in specific cancer or immune cells. The problem is complicated by the high level of cancer and immune cell heterogeneity, the different combinations of S1PR expression, and diversity of S1PR downstream effectors.

S1P may bind other target molecules important for sphingolipid metabolism and signaling. For instance, phosphatases can bind, dephosphorylate approximately half of the intracellular S1P in endoplasmic reticulum, and direct this sphingolipid towards de novo ceramide synthesis during membrane metabolism and recycling [57, 160] (Fig. 5). S1P lyases can also bind S1P and degrade it into phosphoethanolamine and hexadecenal, which can be used for further glycerolipid and phosphatidylethanolamine syntheses [57, 160]. Large amounts of S1P were detected in the circulation where the this sphingolipid forms complexes with high-density lipo-proteins (HDL) [179]. Substantial extracellular levels of S1P are maintained by erythrocytes [180], platelets [181, 182], endothelial cells [183], and various immune and malignant cells [24, 167]. In majority of these cells, S1P secretion is mediated by ATP-binding cassette transporters (ABC-transporter) [180, 184]. S1P gradient, the difference between the intra- and extra-cellular concentrations of S1P, modulates S1PRs expression and represents a novel factor in the regulation of S1P signaling in the immune system and circulation [169, 184].

The proliferation-stimulating effect of the SphK/S1P/S1PR axis is mediated by growth factor network, including MAPK and epidermal growth factor receptor (EGFR) [21, 167, 185]. Various growth factor receptors, including EGFR and VEGFR, were also shown to induce SphK activation, increase the level of S1P production, and transactivate S1P receptors [21, 167, 185]. Aside from EGFR/ERK1/2 [185, 186], S1PR activation influences signaling patterns of various global targets, such as Notch [187], signal transducer and activator of transcription (STAT)3 [23], Akt/mammalian target of rapamycin (mTOR) [188, 189], NF-κB [186, 190], Hippo-YAP pathway [191], and cyclic-AMP responsive element binding protein (CREB) [192]. Cell-, tissue-, and disease-specific expression of S1PR is mediated by coupling to a range of G proteins [193] and/or other receptors (transactivation mechanisms) [21, 57]. S1PRs network interacts with growth factor receptors, including EGFR [167, 185], vascular endothelial growth factor (VEGF) receptors [22, 23], and IGF receptors [194]. Moreover, the SphK/S1P/S1PR axis may be activated by various hormones and cytokines during basic cell growth maintenance, cell differentiation, and metabolic transformations in cancer cells [21, 193]. The mutual transactivation of the network by growth factor effectors provides limitless opportunities to counterbalance apoptosis.

S1P may trigger S1PR-independent mechanisms via binding to other non-traditional receptors, including transcription factors. S1P was demonstrated to induce S1PR-independent activation of TRAF2 [71, 72], although the effect seems cell- and tissue-specific [195]. S1P can also stimulate gene transcription via binding to histone deacetylase 1/2 (HDAC1/2), an epigenetic regulatory enzyme [196]. Activation of endoplasmic reticulum stress and inflammation in keratinocytes was determined to be mediated by S1P binding to the endoplasmic chaperone protein GRP94, recruitment of TRAF2 to inositol-requiring transmembrane kinase/endoribonuclease 1α (IRE1α), and NF-κB signaling. S1P binding to heat shock protein (HSP) 90α was also detected [197]. S1P binds and inhibits ceramide synthase 2 (CerS2), leading to blockade of ceramide (pro-apoptotic effector) synthesis [198]. There may be other not-yet-identified S1P receptors, including some lipid mediators. For instance, myristate, a component of milk fat, was shown to activate pro-inflammatory responses (such as release of TNF-α and induction of COX-2) in colon tissues. Observed effects of myristate were mediated by an unspecified, intracellular target of S1P and were not blocked by S1PR inhibition [163]. Thus, the S1PR-independent effects of S1P are not uncommon, indicating versatility of this sphingolipid signaling.

### Regulation of apoptosis by the SphK/S1P/S1PRs axis

TNF-α-induced effects are not limited to S1P and instead are mediated by a variety of sphingolipids generated during distinct metabolic processes. It has been postulated that TNF-α triggers both pro-apoptotic (ceramide-related) [161, 185] and anti-apoptotic (SphK/S1PRs axis) signaling branches of the sphingolipid network [57, 72, 92]. Activation of apoptosis and autophagy by ceramide has been extensively reviewed elsewhere [147, 161, 199, 200]. A concept of dynamic sphingolipid-based regulation, called a “sphingolipid rheostat”, was suggested to describe a shift towards apoptosis triggered by increased production of ceramide; while a generation of S1P provides a more sustainable cell survival environment and shifts the balance toward anti-apoptotic effects [161, 199, 200].

Ceramide metabolism in normal and cancer cells is regulated by several enzymes, including glucosylceramide synthase, sphingomyelin synthase, ceramide kinase, ceramidases, and SphK [200]. These enzymes define cell life-to-death balance. However, other cell death regulators, including p53, are involved and often provoke unavoidable cell death [200, 201]. A complex relationship between p53 and ceramide has been described, accentuating the importance of ceramide accumulation during activation of stress responses and DNA damage [202]. Notably, ceramide and p53 can trigger signaling effectors upstream or downstream of each other, resulting in sometimes contradictory effects described elsewhere [200, 201].

The SphK/S1P/S1PRs axis is a powerful molecular tool for the regulation of cell survival. The ability of S1P to protect against apoptosis has been well documented in many normal and malignant cell types exposed to pro-apoptotic stimuli, such as TNF-α/Fas ligands [71, 75, 156], serum deprivation [203], ionizing radiation [204], and anticancer drugs [21, 54, 119, 157]. Inhibition of S1P signaling was shown to enhance apoptosis. For instance, treatment of HCC-38 and MDA-MB-468 cells with SphK1 inhibitor PF543 and doxorubicin resulted in synergistic apoptosis-enhancing effects [205]. During carcinogenesis, the SphK/S1Ps axis is highjacked by cancer cells to promote survival. Its role in the development of cancer drug resistance was extensively reviewed and is associated with transactivation of growth-factor networks, stem cells, and other molecular adaptations [21, 54]. Mechanisms of SphK/S1PR involvement in the regulation of TNF-α-induced cell death are complex and sometimes controversial. Sphingolipids trigger signal transduction branching at several different points of the network. There is a possibility that cancer-induced transformation of SphK/S1PRs signaling is responsible for the development of TNF/TRAIL resistance in cancers, although the hypothesis remains untested. Several interactive hotspots (molecular effectors and networks) between DRs and SphK/S1PR networks are discussed below.

### Regulation of inflammation by the SphK/S1P/S1PRs axis

Inflammation is recognized as one of the contributing and promoting factors of carcinogenesis. The SphK1/S1P axis is part of a large signaling network formed by key pro-inflammatory cytokines, such as TNF-α [58, 72, 156], IL-6 [206], IL-1β [81, 207], CCL5 chemokine (regulated on activation, normal T cell expressed and secreted (RANTES)) [128], and others [54, 208]. Bacterial lipopolysaccharide (LPS) was shown to induce SphK/S1P/S1PR3 activation [209], accentuating the potential involvement of sphingolipids during antibacterial responses. The activation of the sphingolipid axis was accompanied by induction of major genes responsible for the propagation of inflammation (COX-2, IL-1β, IL-6, TNF-α, iNOS) [206, 209]. The effect is mediated by a two-way signal-propagating process. Inflammatory responses mediated by COX-2 also required activation of the SphK1/S1PRs axis during progression and resolution of infection [13, 207]. Accordingly, inhibition of S1PR3 by TY52156 resulted in the inhibition of pro-inflammatory gene signaling [209]. Pharmacological SphK1 inhibition (or genetic silencing) also helped to recover the metabolic characteristics of T cells and induced immune antitumor activity [210]. Sphk1 inhibition may improve immunotherapies and stimulate responses to anti-PD-1 and other immune checkpoint inhibitors (ICIs) [211].

Inflammation is a normal immune response by an organism facing infection. Various normal cells may be affected by inflammation and respond to stimulation by cytokines. Sphingolipids are important mediators of normal inflammatory responses in non-malignant cells. Crosstalk between the Fas network and endogenous sphingolipids was observed in various normal cells during pro-inflammatory processes, including osteoclasts from mice with rheumatoid arthritis (RA). Increased level of S1P was associated with osteoclast apoptosis during the development of RA [212]. Furthermore, COX-2, iNOS, prostaglandin E2 (PGE2), IL-1β, and TNF-α signaling pathways activated the S1P network in macrophages during LPS-induced inflammation [75, 93, 208]. S1P mediates various immune responses, including mast cell degranulation, migration of neutrophils, and maturation of lymphocytes [213]. Interestingly, an anti-inflammatory role of SphK1/S1P was also observed [214]. For instance, activation of S1PR2 prevented excessive macrophage recruitment in a peritonitis model in vivo [215], although the effect is macrophage type- and/or pathology-specific [216]. Levels of IL-1β and IL-18 in plasma of wild-type mice were reduced by application of JTE-013 (S1PR2 antagonist) [216]. In SphK1-null mice (*SphK1*^−/−^), SphK1 was found responsible for suppression of LPS-induced neutrophil oxidant production. Binding of SphK1 to JNK resulted in stabilization of JNK and inhibition of JNK binding to the JNK-interacting protein 3 (JIP3). The change of “partners” prevented the activation of nicotinamide adenine dinucleotide phosphate hydrogen (NADPH) oxidase and NF-κB activation, indicating a novel mechanism of anti-inflammatory signaling via SphK1/JNK interactions [217].

**Fig. 6:**
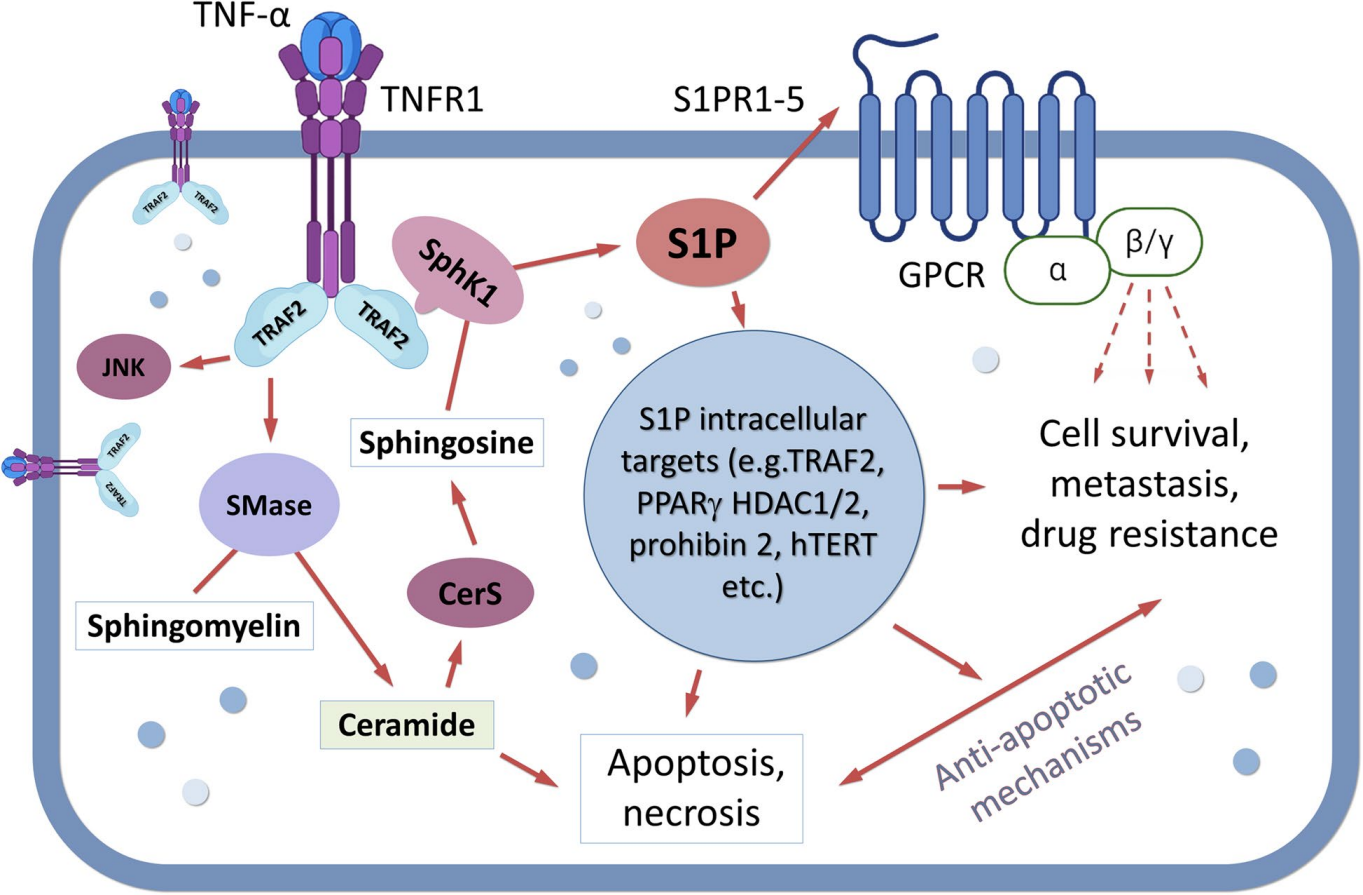
Dichotomy of TNF-α–induced signaling in cancers is hypothetically linked to sphingolipid balance where the relative amounts of ceramide and S1P cause cell proliferation, survival, or death. Stressed cells can increase ceramide in response to TNF-α, resulting in growth arrest and apoptosis. However, in some cells TNF-α can activate SphK and mitigate its pro-apoptotic ability via production of growth-stimulating S1P and activation of S1PR1-5. Abbreviations: CerS, ceramidase; GPCR, G-protein coupled receptor; HDAC1/2, histone deacetylase 1 and 2; hTERT, human telomerase reverse transcriptase gene; JNK, c-Jun NH2-terminal kinase; PPARγ, peroxisome proliferator-activated receptor-γ; SMase, sphingomyelinase

SphK1/2 is involved in the regulation of inflammation in other non-cancerous tissues, though its role is not straightforward. In an in vivo study of arthritis, downregulation of SphK1 decreased inflammation, while total knockdown of SphK2 resulted in a heightened inflammatory response [99]. Similar diversity of the effects of SphK1/2 knockdown was observed during induction of inflammation in the colon [23, 102]. In intestinal epithelial cells, SphK1 was involved in TNF-α/COX-2 pro-inflammatory signaling during exposure to myristate [151]. In neuronal tissue, acetylation of COX-2 via non-specific acetyltransferase activity was also linked to SphK1 activities [218]. Interestingly, triggering of the S1P network resulted in anti-inflammatory effects and suppression of IFN and STAT1 functions [205]. STAT1, a pro-apoptotic effector, controls expression of several cell cycle regulators, enhances death-promoting functions of Bak, and blocks transcription of anti-apoptotic Bcl-2 and Bcl-xL [219]. STAT1 may also induce expression of DR ligands, such as TNF-α, FAS, and TRAIL [45]. Conclusively, limited S1P production via SphK1 knockdown/inhibition may provide an effective tool for a re-activation of the STAT1/IFN pathway [220]. The role of SphK2 in this process remains controversial and should be clarified in future studies. There are reports which indicated contribution of SphK2 in the activation of pro-inflammatory processes [221], which can be (potentially) employed to facilitate anticancer therapies. It is essential to keep in mind the multifactorial role of S1P and provide only cell-targeted reduction of S1P levels and tissue-specific inhibition of SphK1/2 inhibition, as it would be counterproductive to eliminate the effect of sphingolipids on lymphocyte trafficking [54, 193] and anticancer activation. Considering the regulatory role of sphingolipids in T cells, the impact of the SphK/S1P axis should be considered during cancer progression (Fig. 6). The activation of Sphk1/S1PR may significantly change the ability of T cells to recognize and eliminate cancer cells (immunosuppressive effects). To make cancer cells susceptible to T cell recognition/killing, application of nanocarriers and epigenetic reprogramming of malignant cells was suggested as a promising therapeutic approach in this field [222, 223].

### Role sphingolipids in the regulation of lipid metabolism and obesity-associated inflammation

Obesity is regarded as a powerful contributor in the development of cardiovascular diseases and cancer [224, 225]. For instance, obesity-driven inflammation was linked to colorectal cancer progression and metastasis [225]. Low levels of inflammation were found to mark increased fat deposition [226]. Inflammation is promoted in fat tissue by several mechanisms, including imbalanced metabolism, activation of pro-inflammatory immune cells, secretion of cytokines, and other immune mediators [227, 228]. Macrophages and neutrophils located in adipose tissue were shown to secrete pro-inflammatory cytokines (such as IL-1, IL-6, IL-8, C-reactive protein (CRP), TNF-α) [229] (Fig. 3). Accumulation of macrophages in fat tissue and increased secretion of adipokines (fat hormones) were linked to the activation of several signal transduction pathways (JAK/STAT, MAPK, PI3K, mTOR, and 5’AMPK signaling pathways), COX-2 downregulation, and dysregulation of mRNA expression [230]. Excessive saturated fatty acids (SFAs), which are generated in adipose tissues, induce pro-inflammatory signaling in many cell types, including adipose tissue macrophages. SFA deposition also results in enhanced expression of cytokines, such as TNF-α and IL-6 [231]. Interestingly, obesity-related inflammation may trigger carcinogenesis, promote metastasis, and promote cancer immune evasion [229].

The primary function of fat-regulating agents, or adipokines, is to control fat deposition and utilization [232]. Adipokine leptin can suppress appetite by acting upon several mediating effectors, including leptin receptor (*LEPR*) in neurons [233–235]. Surprisingly, cancer cells are also responsive to leptin and express adipokine receptors. Adipokines may activate pro-carcinogenic and metastasis-promoting effects [14, 233]. *LEPR* belongs to class 1 of the cytokine receptor family and is reported to play significant roles in carcinogenesis [236]. It has been shown that leptin induces expression of SphK1 in breast cancer [237]. In another study, leptin-activated SphK1 was demonstrated to trigger IL-6 secretion which maintained low levels of inflammation in the effected tissues [237]. Alternatively, SphK1 deficiency and pharmacological inhibition were associated with adipogenesis, increased expression of regulatory genes associated with adiposity, and production of anti-inflammatory molecules IL-10 and adiponectin. Inhibition of SphK1 resulted in lower recruitment of macrophages and reduced production of TNF-α and IL-6 in adipose tissues [238]. However, sphingolipid regulation of adipose tissue metabolism remains controversial [237, 238].

Interactions between the adipokine network and sphingolipids are delicately balanced by a feedback mechanism of signaling. The role of sphingolipid metabolizing enzymes in adipose tissue has been assessed in several recent studies [151, 239]. SFAs were reported to serve as substrates for ceramide synthases (CerS) and serine palmitoyl transferases (SPT). Both CerS and SPT can modify sphingolipid metabolism [151]. Accordingly, the level of pro-apoptotic ceramide was increased by SFAs (and high fat diet). Moreover, enhanced levels of sphingosine and S1P were found in the blood plasma, liver, and skeletal muscle of rodents following SFA (high fat diet) administration in vivo [237, 239]. In another study utilizing rats, increased expression of SphK2 (but not SphK1) was observed during consumption of fat [240]. However, these studies did not assess the level of pro-inflammatory signaling in those animals, and, therefore, it remains unclear whether these changes led to the propagation of inflammation or just aimed to minimize fat deposition.

The cancer-regulating role of CerS, the dual mediator of adipose tissue effects and sphingolipid metabolizing enzyme, is especially intriguing considering recent findings in breast adenocarcinoma cells. High level of CerS6 decreased phosphorylation of Akt and ERK in MCF-7 breast cancer cells. This effect was associated with inhibition of MCF-7 cell proliferation and activation of the mTOR pathway [241]. The study also analyzed public data using the Cancer Genome Atlas database. Investigators determined the presence of invasive breast carcinoma is negatively associated with CerS6/S1PR2 or CerS6/SphK1 expression. This study suggested that mTOR activity depends on the balance between the production of S1P (by SphK1) and C16-ceramide (by CerS6) [241]. However, it was not tested whether adipose tissue metabolism or adipokines are involved in the regulation of CerS6 and mTOR signaling in breast cancer tissues. The association of these effectors with inflammation and resistance to immunoediting was also not assessed.

A recent study utilized a mice model to show a myristate-enriched milk fat-based diet (MFBD) increased the expression of TNF-α in colonic tissues [151]. MFBD also elevated S1P levels in intestinal epithelium via regulation of SphK1 and JNK [151]. Thus, this data established a link between fat-based diet, activation of SphK1, and increased production of TNF-α (inflammation) in the colon. Further efforts are required to determine whether this condition may potentially result in the inactivation of the anticancer capacity of the TNF network and lead to apoptosis resistance.

Since TNF-α can activate the SphK1/S1Ps receptor axis (and vice versa), it is tempting to hypothesize that this mechanism provides a circuit point which may be essential for internal outcomes of the cell/tissue responses to pro-inflammatory signals. Depending on the existing balance within the sphingolipid network of cancer cells/tissue, the activation of TNF-α/TNFR axis may result in either activation of proliferation (so-called TNF-α resistance mechanism) or apoptosis (traditional death-promoting pathway). The relevant question to ask, what is the 3rd factor(s) that tips the scale of metabolism towards one or another biological process? Considering the role of obesity as a contributing factor in carcinogenesis, adipokines can serve as important contributing factors which may link obesity to advanced cancers and drug resistance. However, high cancer cell heterogeneity (genetic/inherited factors) and the impact of established anti-apoptotic effectors (proteomics and epigenetics) must not be overlooked as powerful contributors.

### The role of sphingolipids in the interaction between ubiquitin-editing enzyme A20 and pro-apoptotic TNF-α signaling

Diverse A20 functions have been linked to dual deubiquitylating enzyme (DUB) and E3-ubiquiting ligase actions [242]. A20 is encoded by TNF-α-induced protein 3 (*TNFAIP3*) gene, a critical anti-inflammatory effector in the TNF network [243]. Anti-apoptotic and cancer stem-cell (CSC) promoting effects of A20 were reported [244]. A20 was defined as an anti-apoptotic and anti-inflammatory effector [245], although A20’s role in the regulation of cancer immune evasion remains largely unclear. For instance, liver regeneration was associated with A20 activities that promoted IL-6/STAT3 pro-inflammatory signaling and suppressor of cytokine signaling 3 (SOCS3) proteolysis [246].

Overexpression of A20 was detected in multiple solid tumors [244], including basal breast cancers with advanced metastatic properties and EMT phenotype [49, 247]. Increased A20 expression in triple-negative breast cancers (TNBC) protected from TNF-α-induced cytotoxic cell death [247]. Lee and co-authors [247] demonstrated that TNF-α induced association of A20 with HSP70, the protein involved in proteolytic removal of damaged and/or incorrectly folded proteins. The formed complex demonstrated increased stability and facilitated resistance to apoptosis in TNBCs, although the effect was not observed in estrogen receptor positive (ER+) luminal cell lines. The failure of TNF-α to trigger A20/HSP70 association in ER+ cells suggested a role for ER in this signaling network [247]. Notably, ER-linked signaling was shown to trigger the SphK1/S1PR axis in ER+ cells (such as MCF-7 cells) [167], while TNF-α was shown to induce apoptosis [153]. Complex and controversial interactions between A20 and estrogen/ER networks were observed [248]. The reported data suggested a potential mutual association between all four effectors (TNF-α, sphingolipids, estrogen, and A20), which remains to be assessed.

A20 was shown to interact with sphingolipid signaling and mediate resistance to Fas/FasL-dependent apoptosis [249]. A recent study indicated that δ-tocotrienol (δTE, a vitamin E form) can stimulate the expression of A20 and inhibit TNF-α-induced activation of NF-κB and LPS-stimulated IL-6 in a concentration- and time-dependent manner in RAW264.7 macrophages [249]. These findings were validated in A20 knockout cells. Treatment with δTE induced generation of dihydroceramides, marked by the activation of cellular stress. Supporting the role of sphingolipid metabolism in A20-dependent effects, myriocin (an inhibitor of de novo sphingolipids synthesis) partially inhibited induction of A20 and A20-induced inhibition of NF-κB by δTE in immune cells [249]. However, this pathway was not tested in cancer cells. Moreover, pro-apoptotic and growth-inhibitory effects of TNF-α were not always associated with the induction of classical NF-κB signaling [250], indicating roles of other genomic and non-genomic mechanisms. A20 was also found to be involved in the regulation of autophagy in T cells [251, 252]. However, less-differentiated (immature) T cells are resistant to TNF-α-induced apoptosis [253]. Considering that T cells express S1PR and are responsive to S1P stimulation [193], it remains to be determined whether the A20/autophagy/sphingolipids signaling mechanism is active in TNF-α-resistant cancer cells and the TME. Supporting the importance of this investigation, sphingolipids were also found to be involved in the regulation of autophagy in different cell types [147, 199].

### NK signaling, TME, and sphingolipids

Human NK cells are a crucial part of the innate immune system responsible for the identification of self/non-self-CD1d (dendritic cells)-presented glycosphingolipids and cytokine-elaborating response [254]. NK cells are cytotoxic towards tumors and demonstrate anti-metastatic properties. Therefore, mutual interactions between TME and NK cells are complex and represent a promising therapeutic avenue for drug development [255]. Tumor cells develop characteristics which allow them to circumvent NK cells, and escape NK-based cytotoxicity. The process is facilitated by chronic stress (hypoxia or ROS) which forces the TME and NK cells to adjust their antitumor functions [256]. The modified TME is immunosuppressive and limits NK cell activity, thus, stimulating tumor progression and spread. NK-mediated resistance was correlated to mutations in DRs/TRAILRs [257]. Anti-apoptotic sphingolipids may contribute this process via their interactions with TNF signaling.

**Fig. 7:**
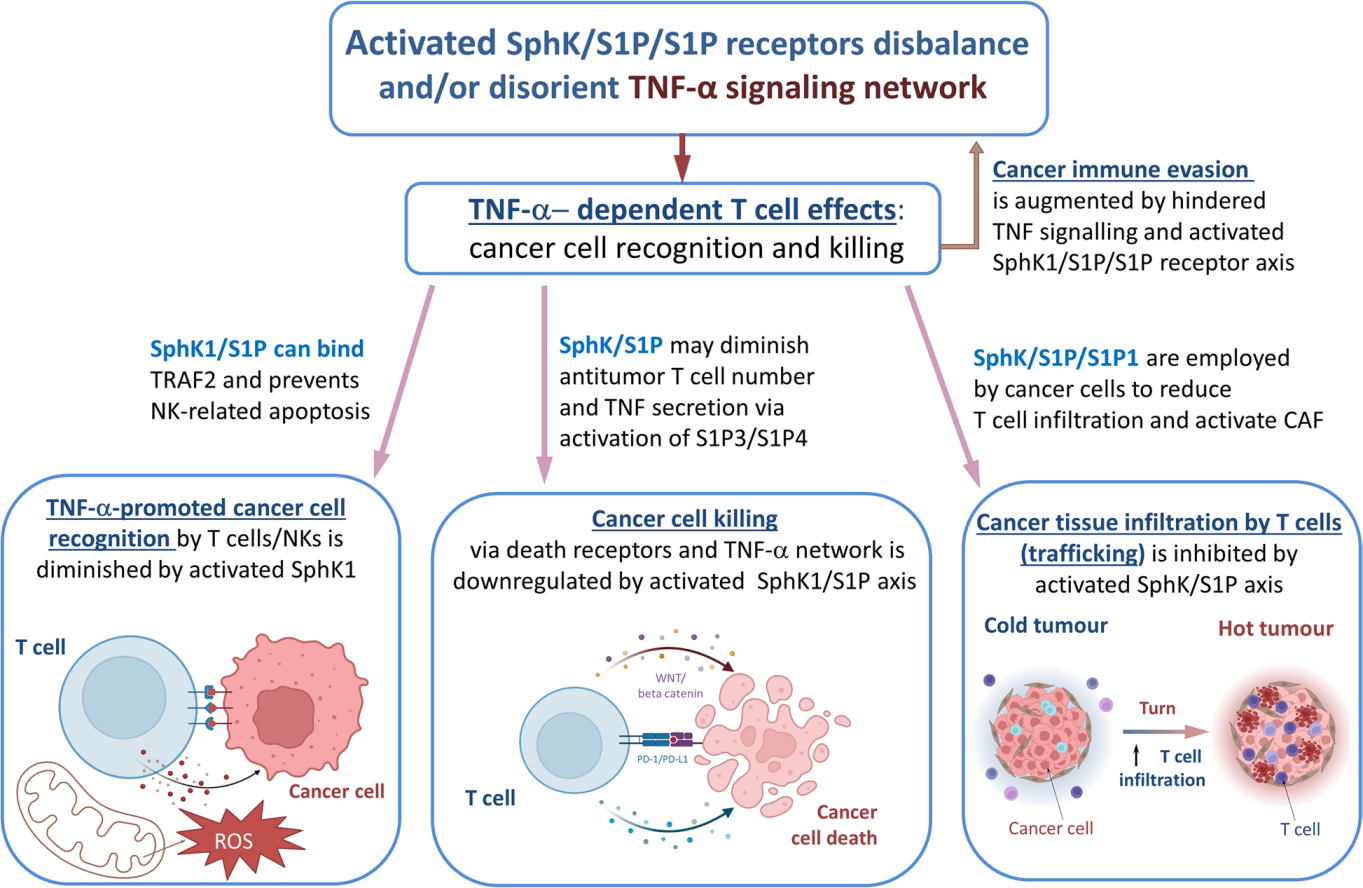
The conceptual model for the regulation of immune T cell responses by the SphK/S1P/S1PR axis during cancer progression. Sphingolipids were shown to impact cancer cell recognition and killing by immune cells at different levels. Abbreviations: PD-1, programmed death-1; PD-L1, programmed death-1 (PD-1) ligand 1; ROS, reactive oxygen species; S1P, sphingosine-1-phosphate; S1P1/S1P3, sphingosine-1-phosphate receptor 1 and 3; SphK, sphingosine kinase; WNT, wingless-related integration site

The list of major regulators of TME/NK responses and biological activities includes the TNF network (Fig.7). For instance, TRAF2 was shown to regulate NK responses [258]. TRAF2 is an adapter protein with E3 ligase properties which binds and activates various signaling molecules, such as membrane-bound receptors, kinases, and phosphatases [242]. TRAF2 can engage E3 ligases, including cIAP1/IAP2, and enable ubiquitination of Complex I components [259]. TRAF2 can be recruited to most proteins in the TNF receptor superfamily and transmits signals to the IKK complex and further to the NF-κB pathway [259]. Recent investigation defined the key role of the cold shock protein Y-box binding protein-1 (YB-1) in the regulation of pro-survival NF-κB p65 signaling by TNF-α via TRAF2. Higher expression of YB-1 was associated with adenocarcinoma invasiveness and expression of CerS6, which regulates cell migration [260]. However, the lower expression of CerS6 was found responsible for enhanced inflammation in a mouse colitis model [261], indicating a diverse role of sphingolipid metabolizing enzymes in the progression of pre-cancerous (pro-inflammatory) and cancerous conditions. As a mediator of TNF-α signaling, TRAF2 has been considered as a potential therapeutic target in cancers. For instance, regulatory T cell (Treg) signaling was targeted by immunotherapeutic approaches which also inhibit TRAF2 [262]. The TNFR2/TRAF2 axis is responsible for co-stimulation of CD8+ T cells, which sensitize cancer cells to cytotoxic effects [41]. TNF-α, was shown to activate Tregs via TNFR2, thus promoting Treg expansion and potential anticancer immunity [11]. However, the role of TNFR2 remains controversial, as both TNFR2 antagonists and agonists have demonstrated anticancer effects [263].

S1P was demonstrated to bind to TRAF2 as a cofactor, changing its E3 ligase biological activity [67, 264]. Blockade of the SphK1/S1P axis resulted in recovery of death-related effects provoked by DR5 knockdown [264]. Generation of S1P was found to be an essential step for TRAF2 polyubiquitination (stabilization), and subsequent promotion of cell invasion [264]. Therefore, TRAF2 is a putative SphK/S1P target during the cancer immune evasion. Binding of S1P to TRAF2, suggestively independent of S1PRs, was associated with activation of ERK1/2 and pro-metastatic cell behavior [264], although this conclusion may require further analysis. Many studies indicated regulation of cell migration by S1P receptors [21, 185]. For instance, S1P receptor 1 (S1P1) transmits S1P effects in various immune cells and regulates egress of lymphocytes into the circulation from the spleen and lymph nodes (LNs) [265]. The binding of S1P (or S1P receptor modulators/ligands) to S1P receptors leads to the receptor internalization and, thus, decreased presence of the receptors on plasma membrane and potential unresponsiveness to future stimulation. The internalization and degradation of S1P receptors may lead to the utilization of intracellular S1P too, which is translated into reduced lymphocyte egress, less circulating lymphocytes, and inhibition of inflammation-linked tissue damage [193]. Accordingly, administration of S1P modulators, such as fingolimod, provoked T cell-targeting immunomodulatory effect indicated by fast decline of the number of blood-circulating T cells [266]. S1P1 also regulates migration of osteoclast precursor cells via Fas/Rac1/NF-κB [219].

Sphingolipid signaling was linked to TME modifications and resistance of cancer cells to NK cell-based elimination [267]. For instance, S1P-stimulated lung cancer-derived monocytes secreted TNF-α and IL-6 in S1P receptor 3 (S1P3)/mTOR/K-Ras-dependent manner, while NF-κB was not implicated [268]. The authors suggested that greater presence of S1P within the TME of lung cancer may orchestrate tumorigenic immune responses [268]. However, this statement requires experimental confirmation, as the exact mechanisms (specific S1P targets) of this effect remain unclear and/or controversial [269, 270]. Furthermore, in non-Hodgkin’s lymphoma, SphK1 silencing resulted in activation of NKs, associated with increased secretion of IL-2 and IFN-γ, which are downstream of the classical NF-κB pathway [271]. Among S1P targets in immune cells, S1P4 (expressed by majority of immune cells) was indicated as a major effector of sphingolipid-dependent effects in innate immunity and lymphocyte trafficking [272, 273]. Therefore, S1P4 signaling could be another potential target to prevent cancer immune evasion. Chemokine CCL2 production by resident macrophages was regulated by S1P4 and synergized with Toll-like receptor (TLR) signaling, indicating sphingolipid receptor involvement in innate immunity responses [274]. Another sphingolipid metabolizing enzyme, S1P lyase, was found to be responsible for suppressing tumorigenicity within the TME [275]. S1P lyase was purported to be a death-promoting enzyme which eliminates S1P and its survival promoting effects [276].

Several independent research groups reported that S1P generation/S1P receptor expression profile stimulates migration of macrophages [277, 278]. Migration toward S1P was found to be mediated by expression of S1P1/S1P3, while expression of S1P2 decreased migration [278]. Interestingly, SphK2 was defined as an anti-inflammatory protein in human macrophages [279], although the role of SphK2 in inflammation remains controversial [221, 280]. Despite a growing number of relevant publications, the physiological roles of secreted and/or intracellularly generated S1P and S1P receptor (subtype-specific) expression in the regulation of macrophage/NK cell migration and activity remain largely unclear. However, the reported S1P-induced activation of human/rodent macrophages by apoptotic cells in an S1P1-dependent manner [278] opens a perspective to use sphingolipids as regulators of chemoattraction in TMEs and potentially increase effectiveness of NK cells. Accordingly, novel S1P receptor modulators and inhibitors require serious testing in vivo [281].

Decreased cytotoxicity of NK cells was associated with changes in chemoattraction and migration of myeloid-derived suppressor (MDS) cells towards tumor tissues [282]. MDS [283], Treg cells, and tumor-associated macrophages (TAMs) are common components of the TME, which can release immunosuppressive cytokines (such as TGFβ) and decrease NK cell-induced apoptosis [284]. In normal tissues, macrophages produce large amounts of TNF-α to clear bacterial and viral infections [285]. However, TAMs are unable to recognize cancer as a tissue destined for clearance, suggesting that the TNF signaling axis is reprogrammed in TAMs. Moreover, low endogenous concentrations of TNF-α derived from macrophages were found to promote metastasis via diverse downstream pro-survival mechanisms [48]. Therefore, TAMs represent a distinct phenotype of macrophages, making them a target for anticancer therapy. Further research is required to define which players within the TNF-α network may be responsible for the cancer-tolerating transformation of TAMs (often defined as de-differentiation) [286] and whether sphingolipids can be targeted in TAMs.

S1P1 and S1P3 represent other promising targets to dimmish cancer immune evasion. S1P1 receptor promoted Treg infiltration and tumor driven Treg expansion in bladder cancer [287]. S1P3 was shown to play a role in modulating the effects of TGFβ in cancer stem cells [288]. These effects were mediated by SphK1 and increased levels of S1P. Similar activation of SphK1 by TGFβ signaling was recently reported in A549 cells [289]. Other anti-apoptotic TME conditions contributed to the blunted immune response and cancer progression. For instance, hypoxia in the TME helps to reduce levels of pro-apoptotic Bax [290] and enhances levels of pro-survival proteins cIAP2 and Mcl-1 [291]. SphK/S1P receptors were shown to be involved in the regulation of this process. SphK2 promoted leukemia cell survival via Mcl-1 [292]. Mcl-1 upregulation was also mediated by S1P1 in mammary cancer cells [293]. Alternatively, fingolimod, an S1P receptor modulator, acted synergistically with TRAIL-induced apoptosis and downregulated Mcl-1 in various human cancer cells [294]. ONO-4641 (another S1P receptor modulator) stimulated the growth of CD11b + Gr-1 + (MDS) cells, decreased T cell proliferation, and lowered INF-γ secretion by CD3+ T cells (with similar characteristics to MDS cells) in the lungs of naïve mice, resulting in the lymphocytopenia [295]. In this mouse model of emphysema, the effect of ONO-4641 was desired [295], although to improve breast cancer immunotherapy depletion of MDS cells should be achieved [288].

The TME also contains non-immune cells (stroma) that promote downregulation of NK cell-mediated effects. Cancer associated fibroblasts (CAFs) are the major component of stroma [288] and confer documented inhibitory effects on NK-cytotoxicity [296, 297]. CAFs were shown to trigger NK cell exhaustion [298, 299] and secrete a range of immunosuppressive cytokines, including IL-10 [300], TGFβ [284], PGE2 [301], and indoleamine 2,3-dioxygenase (IDO) [302]. SphK2 was found to regulate CAF activation via interactions within the p53 network and facilitate the development of cancer tolerance of the TME [303]. Mesenchymal stem cells, which were also observed in stroma, can secrete PGE2/IDO and silence NK cell antitumor effects [301, 304]. S1P1 interaction with IL-22 receptor signaling was found to be involved in the promotion of metastasis to bone by mesenchymal stem cells [305]. Hypoxic conditions are linked to metastasis, inhibition of cancer growth in the initial phase, but promotion of cancer spreading at the later stages. The effects of hypoxia on the TNF-α signaling axis and its association with sphingolipid network are controversial and require further investigations [123, 306].

The controversy relies on the findings that hypoxia can enhance both secretion of pro-inflammatory cytokines (pro-apoptotic effect) and anti-apoptotic hypoxia-inducible factor-1α (HIF-1α) [307]. Hypoxia was also shown to promote resistance of cancer cells to NK cell cytotoxicity [157]. However, there are many network factors involved in the regulation of this process, including expression of HSP90 isoforms [307]. Hypoxia was shown to stimulate S1P generation in HepG2 cells [308] and in ovarian cancer cells [309]. In turn, sphingolipids may regulate hypoxia-related events at different levels. S1P/S1P1, as downstream effectors, mediated HIF-1α signaling during wound healing [212]. Downregulation of SphK1 expression reversed hypoxia-induced cell resistance to NK cell killing via blockade of the S1P/HIF-1α signaling arch [157]. Therefore, silencing or inhibition of SphK1 may be employed to strengthen NK effects. S1P signaling, as an upstream effector, also activated HIF-1α/HSP70 in normal rat pulmonary and cerebral cells [310]. Conclusively, HIF-1α activation by S1P was observed in various cells [311], thus, confirming the hindering effect of S1P in anti-cancer imunity.

The S1P/S1P1-3 axis was found to be involved in the regulation of glucose metabolism in mouse embryonic fibroblasts (S1P lyase knockdown model) via HIF-1α [312]. The S1P2 receptor was reported to be involved in preconditioning of macrophages towards a cancer-hospitable type in the TME [313]. Under hypoxia, a novel sphingosine metabolite O-cyclic phytosphingosine-1-phosphate suppressed mitochondrial dysfunction and apoptosis in mesenchymal stem cells via induction of HIF-1α signaling and calcium-dependent PKCα/ mTOR signaling pathway [247]. S1P modulator fingolimod inhibited HIF-1 and HIF-2 intratumoral levels and sensitized cancer cells to chemotherapy in vivo [314]. The crucial importance of HIF-1 in macrophages is associated with hypoxia-dependent regulation of macrophage interaction with cancer cells and angiogenic potential (interaction with endothelial tissues). Accordingly, strategies to prime macrophages towards anticancer toxicity, attract cytotoxic lymphocytes, and prevent angiogenesis/metastasis using specific inhibitors/modulators of sphingolipid axis (before or together with immune checkpoint inhibitors) could be beneficial.

### Cyclooxygenase-2 (COX-2) and sphingolipids crosstalk

COX-2 is a key enzyme responsible for the production of PGE2, a multifunctional mediator of inflammation, and has been implicated in both inflammation and carcinogenesis. Crosstalk between COX-2 signaling and activation of the PI3K/Akt network has been established. It has been found that the COX-2/PGE2 axis can promote cancer cells survival via PI3K/Akt signaling [315] and Ras-MAPK cascades [316, 317]. Selective nonsteroidal anti-inflammatory drugs (NSAIDs) (such as celecoxib, valdecoxib, and rofecoxib) are widely used to control inflammation and cytokine production [318]. COX-2 is the most studied target of aspirin (the common anti-inflammatory agent), which has also demonstrated anticancer properties [319]. Different COX-2 inhibitors have been suggested as anticancer treatments [317, 320]. Selective COX-2 inhibitors NS-398 and nimesulide have been demonstrated to increase TNF-α sensitivity of TNF-α-resistant HeLa H21 cells [320]. Although nimesulide augmented TNF-α (CD95 or TRAIL receptors)-induced apoptosis, the interaction of TNF-α and COX-2 signaling pathway was not linked to the enzymatic activity of COX-2 [320], and so further analysis is required.

SphK/S1P axis may be involved in COX-2-mediated inflammation via orchestrated interactions with the TNF-α signaling pathway. The S1P receptor-based process seems to rely on both direct COX-2 activation and feedback mechanisms, as TNF-α and other cytokines can trigger SphK1, representing a loop of inflammation-enhancing interactions [213]. S1P-dependent activation of COX-2 was observed in a remarkable variety of normal and malignant cells and tissues, including endothelial [321], and various cancer cells [214, 322]. The SphK1/S1P receptor network also controls PGE2-mediated effects in various cells [81, 323, 324]. Notably, an aspirinyl-conjugated SphK inhibitor (SKI-I-Asp) containing aspirin to bolster oral bioavailability was generated and tested as a promising anticancer drug [325].

S1P effects on COX-2 expression and activity are mediated by its receptors. For instance, S1P stimulated expression of COX-2 and PGE2 production via S1P1 or S1P3 in human granulosa cells [326]. S1P3 antagonist blocked the LPS-dependent induction of COX-2 gene expression [209]. S1P2 mediated inflammation-related effects of S1P in renal cells. However, other enzymes were reported to mediate sphingolipid-induced activation of PGE2 synthesis. SphK1 knockdown decreased cytokine-induced PGE2 production via inhibition of microsomal PGE synthase-1 [322]. It is unclear whether DR expression/signaling is being altered during these effects. In conclusion, tripartite interactions between TNF-α/COX-2/sphingolipid network warrants future investigations.

### DRs cross talk with PKC, STAT1, and SphK1/S1P3

Akt is a serine/threonine kinase which can be activated downstream of PI3K to provide a critical defense against apoptosis [327]. Activated Akt/PI3K can phosphorylate many mediators of DR signaling, including caspase-3 and caspase-9, Bad, MDM2, and different transcription factors [293, 328, 329]. In TNF-α-treated breast cancer cells, PKCε mediated anti-apoptotic effects via direct association with Akt [330–332]. Aside from anti-apoptotic ERK1/2 [98], the PKC/Akt axis also mediates sphingolipid effects [333]. For instance, PKC was found to be involved in S1P-mediated calcium fluxes and induction of insulin secretion in pancreatic β cells [334]. PKC has been reported to mediate the activation of endothelial cell migration and signaling [189, 335], and survival of malignant cells [336]. Akt activation by S1P, which mediated resistance to ischemia/reperfusion injury, was also reported in endothelial cells and cardiac myocytes [188, 337]. S1P3 was found to be involved in stabilization of Akt mRNA and stimulated Akt protein expression [338]. PKC/Akt may mediate poor response to immune checkpoint blockade therapy [339], and, thus, inhibition S1P axis may be beneficial in less responsive patients [340]. Interestingly, S1P2 was reported to mediate PKC inhibition [341].

Contrary to the S1P/S1P3 receptor network, ceramide and sphingosine may serve as negative regulators of PKC/PI3K/Akt signaling via several potential mechanisms [342–344]. Binding of PKCζ to 14-3-3 scaffolding proteins was found to be disrupted by ceramide, leading to PKCζ recruitment to lipid rafts [344]. Ceramide can also regulate Akt translocation to the plasma membrane and redirect (or block) its anti-apoptotic effects [343]. Ceramide-induced negative regulation of growth was marked by decreased ERK activity through PKCε-dependent effects [342]. PKCε was blocked by ceramide which prevented PKCε binding to Raf-1 and ERK in cells treated with insulin-like growth factor [342].

The PKC/Akt axis is a key regulator of autophagy [345] which can be activated by C2-ceramide in cancer cells [346–348]. SphK1 was also found to be activated in starved cells [349]. However, the role of SphK1 and S1P in the regulation of autophagy remains controversial and may be independent of Akt signaling [350]. Moreover, SphK1 activation during starvation may be a result of inducible cytoprotective mechanisms. This suggestion is supported by a study which indicated that SphK1 downregulation by siRNA enhanced starved cell death [350]. The involvement of the sphingolipid axis in the TNF/TRAIL-induced cell death may be also more complex than it was originally anticipated. However, the role of SphK/S1P axis in the regulation of autophagy in immune cells warrants further investigation, considering that autophagy is a promising target to overcome resistance to immunotherapy [351].

Stimulation of proliferation and anti-apoptotic effects of PKCε were mediated by a network which includes not only ERK1/2 and PI3K/Akt, but also STAT1, STAT3, and NF-κB pathways [352]. The potential role of putative STAT1 sites in the regulation of PKCε transcriptional activities was tested in MCF-7 cells [353]. The study demonstrated involvement of STAT1 and Sp1 in the upregulation of PKCε in MCF-7 cells in vitro [353]. The interaction is also a two-way mechanism, as inhibition of classical PKC isoenzymes resulted in downregulation of STAT1 in macrophages [354]. STAT1 was shown to regulate mammary tumorigenesis via multiple effectors [45, 219, 300]. SphK1 was reported to suppress activation of STAT1 in both parental and breast CSC cultures [220]. Another recent study indicated that STAT1 may bind the promoter region of S1P1 receptor [355]. It remains to be discovered how the anti-apoptotic effects of Akt/PKC can be integrated with STAT1 and sphingolipid networks in cells resistant to TNF-α/TRAIL-induced apoptosis.

## Exploring the role of natural dietary and plant-based compounds as regulators of inflammation and sphingolipid metabolism

Selective anti-inflammatory molecules, including natural plant compounds and dietary components, have been shown to impact activation of pro-apoptotic cytokine signaling, suggesting their potential as safe and efficacious options for drug-resistant tumors. For instance, sulforaphane (SFN), a dietary component of broccoli, is an effective antioxidant with anticancer and anti-inflammatory characteristics [356] that has been tested for its cancer chemo-preventive properties [357–359]. Cytoprotective effects of SFN were associated with induction of the Nrf2 signaling pathway [357]. Alternatively, SFN-induced downregulation of Nrf2 expression was linked to increased apoptosis and elevated ROS [360]. SFN reversed ceramide-mediated apoptosis [361] in mouse hepatocytes that resulted from a high-fat diet (HFD) via the Nrf2 pathway [361]. Involvement of the Nrf2 pathway was also observed during application of siponimod (BAF312), a selective modulator of S1P1 and S1P5 receptors [362], supporting the existence of connections between Nrf2 and the sphingolipid signaling network. Siponemod demonstrated anti-inflammatory properties and microglia-protecting effects in the brain [363]. These effects provide insight into the regulation of S1P receptor signaling during inflammation, although the immune re-activating effects of these agents remains to be tested.

Natural flavonoids can regulate redox-sensitive pathways and transcription factors (such as Nrf2 and NF-κB) associated with increased release of free radicals/ROS and chronic inflammation [358, 364]. Many natural compounds were also found to target TNF-α/NF-κB and DR5 expression/pathway in cancer cells [365]. However, the effect of natural compounds on sphingolipid and TNF signaling networks during cancer immune evasion remains largely unclear. Only some of plant-derived and dietary compounds were tested and reported to influence sphingolipid metabolism and/or TNF network activity. One of the most studied agents, apigenin (4’,5,6-trihydroxyflavone), an anti-inflammatory compound isolated from parsley, oranges, and other plants, demonstrated strong anticancer properties via regulation of TNF-α, and DR4/DR5 pathways [35, 366, 367]. In conjunction with TNF-α, apigenin was shown to stimulate apoptosis and effectively decreased the survival of colon cancer cells [367]. In HepG2 cells, apigenin stimulated apoptosis via activation of pro-apoptotic TNF-α signaling [368]. Sensitization to Apo2L/TRAIL-induced apoptosis was also reported in prostate [369], HepG2 [370], Huh-7 (HCC) [371], and lung cancer cells [372] treated with apigenin. This dietary compound induces NF-κB activation [373]. Upregulation of TNF-α synthesis by apigenin was observed in J774.2 macrophages [374]. Notably, apigenin was also shown to inhibit SphK1/S1P axis in cardiac cells during endotoxemic shock [136]. However, in breast cancer cells, a dual effect of apigenin fostered some doubts about clinical application of this agent. Low doses of apigenin stimulated cancer cell growth, while high doses activated apoptosis via the TNF-α pathway [375]. Controversial findings were also reported in RAW264.7 macrophages where apigenin inhibited the effects of TNF-α [376]. Accordingly, detailed investigation is warranted to confirm the anticancer and SphK1/S1P-inbiting effects of apigenin in resistant tumors.

Other promising anti-inflammatory and SphK1-inhibiting agents (phenols and polyphenols) capable of sensitizing cancer cells to the pro-apoptotic effects of TNF-α (and/or stimulate pro-apoptotic effects of TRAIL/DR signaling) include the flavonoid epigallocatechin gallate (EGCG) [377] and the polyphenol resveratrol [378–380]. Protective effects of EGCG gavage were associated with increased levels of immune-enhancing substances. The agent also helped to balance regulation of the serum levels of sphingomyelin and sphingomyelin in the LPS-induced acute injury models, leading to reduced effects of harmful substances and inflammation [381]. Resveratrol was shown to impact sphingolipid metabolism in lung adenocarcinoma cells and downregulate inflammation via SphK1 inhibition [101, 380]. Another flavonoid, quercetin, also demonstrated antioxidant properties and reduced the production of S1P in HepG2 cells [382]. However, further testing is required to determine the immunomodulatory effects of dietary compounds in patients with resistant cancers.

## Future perspectives of TNF-α/TRAIL therapy and clinical application of agents targeting the sphingolipid pathway

Major immunotherapies aim to increase the amount of tumor antigen-specific effector T cells in the circulation, block immunosuppressive effects of the TME [48], and stimulate cancer cell-targeted inflammation. The decision to initiate immunotherapy should be made on a per-patient basis according to the expression of predictive biomarkers (“immune response” gene signature). Several recent clinical trials have tested recombinant human TRAIL (rhTRAIL) and TRAIL receptor agonists (TRAs) against TRAIL-R1 and TRAIL-R2 [2]. DRs/TNFR1 have been the target of monoclonal antibodies (mAbs) in clinical trials over the last decade with variable levels of success [47]. Recent trials indicate high mAb specificity, longer half-life, and fewer adverse effects compared to conventional treatment [383]. TNF-α-containing fusion proteins have been designed and show effective anticancer properties [384]. However, only gene therapy with VB-111 (ofranergene obadenovec) yielded significantly improved progression-free survival in one trial [385], while another failed to confirm its efficacy in combination with bevacizumab (phase III study: NTC02511405) [386]. VB-111 was constructed using a replication-defective adenovirus serotype 5 vector attached to a modified murine pre-proendothelin promoter (PPE-1) and human Fas-chimera transgene [387]. Current data indicates that sphingolipids contribute to the development of cancer resistance to both immune surveillance and TNF/TRAIL-induced apoptosis, representing a promising target for future clinical strategies. The addition of sphingolipid modulators may increase the efficacy of this treatment, although this hypothesis is yet to be tested.

Several decades ago, the glycolytic pathway was suggested as a clinical target to sensitize tumor cells to soluble death ligands [52]. Glucose deprivation or inhibition of glucose metabolism enhanced apoptosis induced by TNF-α, CD95 agonistic antibody, and TRAIL in myeloid leukemia U937, cervical carcinoma HeLa, and breast carcinoma MCF-7 cells [52]. The effect was also observed in the human B-lymphoblastoid cell line SKW6.4, a prototype line for mitochondria-independent DR-induced apoptosis. Changes in c-FLIP(L) and cFLIPs levels were observed in some but not all studies cell lines under glucose deprivation [52, 388]. The changes were associated with activation of mitochondrial metabolism [388, 389]. Recent findings indicate a key role of sphingolipids in the regulation of cancer metabolism and anticancer immune responses [20, 390]. Dramatic changes in sphingolipid composition and processing were reported in cancer tissues [391]. Considering the involvement of sphingolipid network in TNF-α/TRAIL-activated signaling, it is reasonable to test SphK1/S1P receptor axis modulators as substances capable of strengthening anticancer therapy and increasing overall survival.

The delivery of TME-stimulating agents and reprogramming of the TME can be facilitated by nanocarriers [222, 392]. It has been shown that localized delivery of a nanoparticle-conjugated TLR7/8 agonist triggered lymph node-located DCs activation and promoted proliferation of tumor antigen-specific CD8+ T cells [392]. Cancer cell-targeted delivery of complex death-enhancing agents has demonstrated promising preclinical results. TRAIL-anchored artificial liposomes (defined as large unilamellar vesicle (LUV)) were constructed and loaded with DOX (named as LUVDOX-TRAIL). The liposome nanoparticle permitted synergistic cytotoxic potential compared to the effects of DOX or TRAIL alone. LUVDOX-TRAIL cytotoxicity was associated with faster internalization of the DOX-loaded liposomes and TRAIL-induced activation of caspase-8 [393]. Manipulation of tumor ceramide (and/or ceramide-conjugate substance) levels was explored as a potential strategy against drug resistant breast cancers [394, 395]. Some original studies have utilized the structurally modified analogs of the sphingoid backbone of d-erythro-N-octanoyl-sphingosine (Cer). The most potent anti-proliferative analog (2S,3R)-(4E,6E)-2-octanoylamidooctadecadiene-1,3-diol (4,6-diene-Cer) induced apoptosis in TNF-α-resistant MCF-7 cells, MDA-MB-231, and NCI/ADR-RES breast cancer cell lines [395]. Detected death-related mechanisms of 4,6-diene-Cer included a prolonged elevation of intracellular Cer and were mediated by the mitochondrial apoptotic pathway. Moreover, the valuable clinical characteristics of 4,6-diene-Cer include selectivity toward transformed breast cells [395]. Although the original substances turned out to be quite toxic in vivo, the search for less toxic substances continues. It has been found that 3-ketone-4,6-diene ceramide efficiently kills chemo-resistant breast cancer cells [396]. Recently, new ceramides with anticancer properties were extracted from red algae of the Red Sea [397].

Several novel methods were designed to deliver TNF-α locally as part of intratumoral vaccination [398]. The efficient nonviral gene therapy was developed to provide localized transfer of multiple genes into tumors in vivo. Gene electrotransfer (GET) was named as the most efficient method of local delivery of toxic cytokines. For instance, TNF-α and IL-12 (both can boost the primed local immune response) genes were transferred in murine melanoma cancers using GET [399]. The transfer was followed by a pronounced delay in tumor growth associated with strong antitumor immune response with extensive infiltration of immune cells in the tumor site [400]. Notably, GET was accompanied by resistance of the mice to secondary challenge with tumor cells [399]. Furthermore, phage and yeast display (bacteriophage strategy) were used for a pre-selection of non-neutralizing antibodies which were used to “piggyback” on TNF-α and enter cells through binding TNFRs [401]. This approach successfully reshaped the TME towards recruitment of antitumor immune cells (such as N1 neutrophils, M1 macrophages, and activated CD4+/CD8+ T cells) [401]. Combined testing of this bacteriophage technology with SphK1/S1P-targeting agents warrants future investigation.

Several conserved TNF-derived peptides can trigger apoptosis and/or necrosis in tumor cells [402] independent of TNFRs. Some of the necrosis-inducing TNF-derived peptides (like P1516) with strong membrane-disrupting characteristics may be released during TNF degradation [402]. The peptide’s cytolytic property was linked to its unique β-barrel/β-hairpin secondary structure [3]. Immunohistochemical analysis of tumor tissues from P1516-treated mice indicated extensive destruction to the cancer vasculature [402], which was associated with lower metastasis and better survival. The study indicated that TNF sequence contains cryptic functions that are triggered only after TNF partial and or specific degradation. This finding opens a previously unexplored perspective of TNF biology relevant to immune regulation and cancer immune surveillance. TNF-derived peptides P15 and P16 were suggested as a novel class of antitumor agents [402].

Tumor-specific cytotoxic T lymphocytes (CTLs) represent a natural and highly effective tool in cancer immunotherapy [4]. Considering the immunoregulatory role of sphingolipids (specifically the SphK1/S1P receptor axis), agents targeting sphingolipids may be employed to manipulate CTLs. However, only very few agents targeting this pathway have been approved for anticancer clinical testing. The approved agents include fingolimod (an S1P receptor antagonist; Phase I; NCT02490930 and NCT03941743); Safingol (L-threo-dihydrosphingosine; a PKC inhibitor; Phase 1; NCT01553071 and NCT00084812); sonepcizumab (ASONEP; an S1P-specific monoclonal antibody; Phase I/II; NCT00661414 and NCT01762033); ABC294640 (an SphK2 inhibitor; Phase I/II; NCT01488513, NCT02229981, NCT02757326, NCT02939807, NCT03377179, and NCT03414489). One study is currently recruiting to assess sphingolipids as predictive biomarkers in melanoma (NCT03627026). However, the potential testing will be on the way when new SphK1/S1PR modulators/inhibitors are generated and evaluated in preclinical settings. As a promising sign, the synergistic effects of DOX and SphK1 inhibition were reported in breast cancer cells [220].

A novel and less toxic strategy for advanced T cell infiltration in cancers has been suggested recently. A fusion protein Cys–Asn–Gly–Arg–Cys–Gly–TNF (called NGR-TNF) capable of targeting the cancer vasculature was constructed by Elia et al. [403] to assist intratumor infiltration by activated CTLs. It has been reported that, in a transgenic prostate adenocarcinoma mice model, combined treatment with NGR-TNF (with adoptive T cell therapy (ACT) and immune checkpoint blockade) effectively improved overall survival and delayed the disease progression. NGR-TNF promoted tumor infiltration by CTLs associated with beneficial T-effector/Treg cell ratios [403]. The authors of this study suggest that therapeutic targeting of sphingolipid pathway may contribute to this process.

## Conclusions

Despite all the therapeutic impediments of TNF-α/TRAIL application, the cytotoxic cytokines remain the strongest natural defense to cancer in humans. TNF-α to be a prominent effector of immune surveillance which can kill mutated or abnormal cells, including cancer cells, under physiologic conditions [37]. Thus, to improve current therapeutic methods, it would be beneficial to preserve the pro-apoptotic capacity of TNF-α and block only its pro-survival branch. Notably, the cancer-promoting chronic inflammation which contributes oncogenic transformation, underscores a need to decipher the DR pathway and design agents that will block TNF/TRAIL/DR pro-survival signaling [401]. A wide range of substances and therapeutic methods has been developed to enhance immunotherapy effects in cancer patients [394, 396–399, 401–404], although combined application of sphingolipid-targeting agents and TNF-α pathway activating methods seems neglected. Apparently, SphK/S1P/S1PR axis plays an important role in transduction of TNF-α effects, both as a mediator and regulator of the cytokine signaling (Table 1). The generated anticancer agents which can selectively inhibit the growth-promoting effects of SphK (including dual SphK isozyme inhibitor, SKI-II (4-[4-(4-chloro-phenyl)-thiazol-2-ylamino]-phenol) represent a class of promising therapeutic substances [119, 405]. However, the production of agents that target the proper SphK isoform in cancer cells is challenging, although a large group of patented agents has been synthesized [406].

Nanoparticles represent a very promising approach for the targeted delivery of immunotherapy agents. Among the cutting-edge nanomedicine vehicles is a group of artificial liposomes with anchored sTRAIL, called LUV-TRAIL, which also improved delivery and reduced toxicity of immunotherapy [393]. Recent studies have tested the delivery and anticancer effects of TNF-α-loaded liposomes [404] or plant viral nanoparticles [407], TRAIL/paclitaxel multifunctional nanocarrier, graphene-based nanocarrier with DR4-targeting antibody/AKT siRNA, and anti-DR5-conjugated lipid-based nanocarriers [408]. Other nanoparticle-based agents displayed efficient pro-apoptotic properties via interactions with DR-signaling, including CD95 receptors [409] and the TRAIL network [400]. However, most of these studies were conducted in vitro, indicating a need for additional in vivo experiments before clinical testing may be considered. The addition of sphingolipid modulators to this regimen, specifically novel inhibitors of S1P1-S1P3 receptors, may augment the efficacy of nanoparticles in future studies. The success of personalized immunotherapy towards the re-activation and/or reformation of natural anti-cancer immunity may be defined by the de-activation of SphK/S1P/S1PR axis using novel inhibitors of sphingolipid pathway.

## Data Availability

Not applicable.

## Abbreviations

4,6-diene-Cer: (2S,3R)-(4E,6E)-2-octanoylamidooctadecadiene-1,3-diol
ACE: Angiotensin converting enzyme
Apaf-1: Apoptosis protease activating factor-1
ATF4: Protein kinase R-like endoplasmic reticulum kinase-mediated activating transcription factor 4
Bak: Bcl-2 antagonist killer 1
Bax: Bcl2 associated X protein
Bcl-2: B-cell lymphoma-2 protein
BMM: Bone marrow-derived monocyte/macrophage
C1P: Ceramide-1-phosphate
CAFs: Cancer associated fibroblasts
CDase: Ceramidase
Cer: D-erythro-N-octanoyl-sphingosine
CERK: Ceramide kinase
CerS: Ceramide synthase
CERT: Ceramide transport protein
CFTR: Cystic fibrosis transmembrane conductance regulator
CGA: Chlorogenic acid
cIAP: Cellular inhibitor of apoptosis protein
COX-2: Cyclooxygenase-2
CRDs: Cysteine-rich domains
CREB: Cyclic-AMP responsive element binding protein
CSC: Cancer stem-cell
CTLs: Cytotoxic T lymphocyte
cyt *c*: Cytochrome *c*
DcR: Decoy receptor
DD: Death domain
DED: Death effector domain
DENV: Dengue virus
DHS: Dihydroxysphingosine
DIABLO: Direct Inhibitor of Apoptosis-Binding protein with Low pI
DMS: N,N-dimethylsphingosine
DUB: Dual deubiquitylating enzyme
EGCG: Epigallocatechin gallate
EMT: Epithelial-to-mesenchymal transition
eNOS: Endothelial nitric oxide (NO) synthase
ER: Estrogen receptor
ERK: Extracellular-signal-regulated kinase
EZH2: Enhancer of zeste homolog 2, a histone methyltransferase
FADD: Fas-associated death domain
FLICE: FADD-like IL-1β-converting enzyme
FLIP: FLICE inhibitory protein
fMLP: N-Formylmethionyl-leucyl-phenylalanine
GET: Gene electrotransfer
GFAP: Glial fibrillary acidic protein
HCC: Hepatocellular carcinoma
HDAC: Histone deacetylase
HFD: High-fat diet
HIF-1α: Hypoxia-inducible factor-1α
HMVEC-C: Human cardiac microvascular endothelial cells
HSP: Heat shock protein
ICIs: Immune checkpoint inhibitors
IDO: Indoleamine 2,3-dioxygenase
IFN-γ: Interferon-γ
IGF: Insulin-like growth factor
IKK: I-kappa-B kinase
iNOS: Inducible nitric oxide synthase
IRE1α: Inositol-requiring transmembrane kinase/endoribonuclease 1α
JAK2: Janus kinase 2
JIP3: JNK-interacting protein 3
JNK: C-Jun N-terminal kinase
LAMP-2: Lysosomal associated membrane protein-2
LEPR: Leptin receptor
LPS: Lipopolysaccharide
LUV: Large unilamellar vesicle
mAbs: Monoclonal antibodies
MAPK: Mitogen-activated protein kinase
MDS: Myeloid-derived suppressor
MFBD: Myristate-enriched milk fat-based diet
MMP: Matrix metalloproteinase
MnSOD: Mitochondrial superoxide dismutase
mTNF-α: Transmembrane TNF-α
mTOR: Akt/mammalian target of rapamycin
mtROS: Mitochondrial reactive oxygen species
NADPH: Nicotinamide adenine dinucleotide phosphate hydrogen
NF-κB: Nuclear factor kappa B
NGR-TNF: Cys–Asn–Gly–Arg–Cys–Gly–TNF fusion protein
NK: Natural killer T cells
NSAIDs: Nonsteroidal anti-inflammatory drugs
OPG: Osteoprotegerin
OPGL: Osteoprotegerin ligand
OXPHOS: Oxidative phosphorylation
PGE2: Prostaglandin E2
PI3K: Phosphatidylinositol 3-kinase
PPE-1: Pre-proendothelin promoter
RANTES: Regulated upon activation, normal T cell expressed and secreted (CCL5)
rhTRAIL: Recombinant human TRAIL
RIP: Receptor interacting protein
RIPK1: Receptor-interacting protein kinase 1
RTKs: Receptor tyrosine kinases
S1P: Sphingosine-1-phosphate
S1P1: S1P3, sphingosine 1-phosphate receptor 1 and 3
S1PR: S1P receptor
SFAs: Saturated fatty acids
SFN: Sulforaphane
siRNA: Small interfering ribonucleic acid
SKI-I-Asp: Aspirinyl-conjugated SphK inhibitor
SKI-II: 4-[4-(4-Chloro-phenyl)-thiazol-2-ylamino]-phenol
Smac: Second mitochondria-derived activator of caspase
SOCS3: Suppressor of cytokine signaling 3
SphK: Sphingosine kinase
SPL: S1P lyase
SPPase1: Sphingosine-1-phosphate (S1P) phosphatase 1
SPT: Serine palmitoyltransferase
STAT: Signal transducer and activator of transcription
TAM: Tumor-associated macrophage
TGFβ: Transforming growth factor β
TL1A: TNF-like cytokine 1A
TME: Tumor microenvironment
TNFAIP3: TNF-α-induced protein 3
TNFR: TNF-α receptor
TNF-α: Tumor necrosis factor-α
TRADD: TNFR-associated death domain
TRAF2: TNF receptor-associated factor 2
TRAIL: TNF-related apoptosis-inducing ligand
Treg: Regulatory T cell
VCAM-1: Vascular cell adhesion molecule-1
VEGF: Vascular endothelial growth factor
YB-1: Y-box binding protein-1
δTE: δ-Tocotrienol
